# A Golden Jackal based optimal operation and optimization of a virtual power plant involving renewable resources

**DOI:** 10.1038/s41598-025-09164-y

**Published:** 2025-07-07

**Authors:** Anubhav Kumar Pandey, Chaima Mansour, Nandini K. K., Vinay Kumar Jadoun, Jayalakshmi N. S.

**Affiliations:** 1 Department of Electrical and Electronics Engineering, Manipal Institute of Technology, Manipal Academy of Higher Education, Manipal, Karnataka 576104 India; 2 Department of Electrical and Electronics Engineering, Dayananda Sagar College of Engineering, Bengaluru, Karnataka 560078 India; 3 Department of Energy, National School of Engineer of Tunis, Street Béchir Salem Belkhiria Campus University, Le Belvédère, BP 37, 1002 Tunis, Tunisia; 4 Department of Electrical and Electronics Engineering, NMAM Institute of Technology, Nitte (Deemed to be University), Nitte, Karnataka 574110 India

**Keywords:** Energy management, Metaheuristic, Optimization, Renewable sources, Virtual power plant, Electrical and electronic engineering, Energy grids and networks, Power distribution, Solar energy, Wind energy

## Abstract

This paper reports the optimum operation of a virtual power plant (VPP) to reduce the reliance on coal and gas-based energy generation. This practice not only promotes the incorporation of renewable-based energy sources but also reduces the reliance on fossil-based resources which are limited in the reserves. The selected VPP system comprises small hydropower, wind turbine, solar photovoltaics and fuel cells accompanied by a co-generation unit. The proposed VPP is also equipped with a storage facility applicable as a flexible storage option i.e., an electric vehicle followed by an energy storage system. In addition, the primary objective of this work is to lessen the overall cost associated with the system along with a reduction in the generated emissions. Moreover, a Golden Jackal-based Optimization inspired by nature is utilized in a single and multi-objective framework to attain the desired target objectives. Furthermore, the scheduling of the VPP system is carried out on a day-ahead basis and the outcome in terms of cost and emission is compared with the available work in the literature and the result reveals the effectiveness of the system in terms of quality solutions sets for the objectives, minimum computational time and better convergence behavior. In particular, there is a reduction in cost by 2.1% and 0.62% when compared with the salp swarm algorithm and beluga whale algorithm followed by 2.12% and 1.18% in emission. Furthermore, the cost/emission of the VPP system is also abridged by employing a Pareto-based approach which shows the suitability and effectiveness of the developed system.

**Supplementary Information:**

The online version contains supplementary material available at 10.1038/s41598-025-09164-y.

## Introduction

Historically, the reliance on non-renewable and conventional powered energy resources has been crucial to fulfill our energy needs. Fossil fuels generally power these resources or gas-fired power plants that have played a significant role in powering industries, transportation sectors, and residential premises. However, the environmental consequences associated with their extraction, production, and combustion cannot be ignored. Nonetheless, there are viable strategies to mitigate these environmental impacts. One key approach is to decrease our dependence on non-renewable resources by utilizing them less frequently. This can be achieved through the widespread adoption of renewable energy resources (RERs) by encouraging energy production and consumption from environment-friendly sources viz., solar, wind, hydro, and geothermal power. These clean and renewable alternatives offer a sustainable pathway to meet our energy needs while minimizing the harmful pollutants to the environment. Furthermore, incorporating optimization techniques to leverage the productivity of systems can yield substantial benefits. By exploring innovative technologies, process improvements, and technologically advanced energy management systems (EMS), we can optimize resource usage across various sectors specifically in the energy domain. These optimizations not only contribute to environmental conservation but also present economic advantages. By reducing resource consumption, VPP can lower operational costs, enhance efficiency, and maximize overall profits. In addition to reducing usage and integrating RERs, another avenue that is often ignored is minimizing the environmental impact of non-renewable resources by implementing a Virtual Power Plant (VPP)^1^. Unlike traditional power plants, a VPP is operated independently and can be interconnected to perform combined and holistic operations by using advanced software for decentralized control and monitoring^2^. However, a VPP doesn’t generate or own energy itself, it efficiently aggregates power from multiple sources and operates as a single entity which can ensure consistent power flow and maintain the balance between demand and supply. The VPP also helps in reducing aggregated technical and commercial losses, decreasing emissions, and providing monetary benefits to its participants. It plays a crucial role in integrating heterogeneous RERs like solar and wind power, mitigating their production fluctuations, and maintaining grid stability in real-time management and emergency situations.

### Motivation

Several challenges exist in ensuring the optimal operation of the energy infrastructure to maintain a balance between demand and supply during operational hours. One of the major obstacles is the lack of real-time data availability, making it difficult to accurately forecast the demand and execute efficient demand response (DR) strategies. Accurately predicting demand is crucial for effectively scheduling and managing the VPP system. To overcome these challenges, the energy sector is experiencing a significant boost, allowing households to generate electricity and contribute surplus electricity back to the national grid. A VPP is a cloud-based energy hub comprising a decentralized power source that brings together various distributed energy resources (DERs) from multiple sites to optimize power generation and consumption by actively participating in electricity markets. In the case of electric vehicles (EVs) which are considered as a soft-load can be arbitrarily equipped to a VPP as they can fulfill demand in peak loading by sending the power supply back to the grid called as a vehicle-2-grid (V2G) and they can be exploited as a storage system during its unutilized period either in the residential premises or in the parking lot during office hours. As a result, ensuring optimal scheduling of power becomes crucial, not only for minimizing the cost and enhancing efficiency but also for effective functioning and management of energy resources.

### Related work

In this section, the studies carried out regarding the optimal scheduling of VPP are compiled by highlighting the related work performed. Yang et al.^3^ presented a study of a multi-objective allocation and scheduling for a VPP composed of a single renewable energy (RE) source: WT and EV in parking lots. In this study, the authors utilized a golden jackal-based optimization based on the strategy of Rosenbrock’s direct rotational (RDR) to determine the size as well as the location of their chosen resources that give them an optimized cost, battery degradation cost, and voltage deviations during 365 days. The approach claimed to have better performances compared to other methods. The demerit of this work is it didn’t include a lot of resources, so the power-generated indices wouldn’t be so optimal. Wang et al.^4^, studied a VPP composed of V2G mode by integrating PEVs, micro turbine (MT) and renewable-based energy sources i.e., PV and flexible loads. The case study used the day ahead (DA) market revenue and the costs brought about by real-time (RT) market deviations, and three strategies were compared. Finally, by increasing the number of PEVs, the solar energy accommodation of the VPP is enhanced by nearly 0.6% on an annual basis. Niknam et al.^5^, considered the uncertainties that arose from renewables and the varying load demand along with the fluctuation in market price using the 2 m (PEM). The second objective is the optimization of energy management in which the total operating cost for a microgrid (MG) is optimized using a gravitational search-based algorithm (GSA). The microgrid comprises wind turbines, PV and fuel cells FC, and ESS. The paper proposed two different methods to progress the convergence characteristics obtained from GSA. In the first method, the authors employed the GSA as a memory-less algorithm so that the particles do not use the appropriate information found in earlier iterations. This is followed by a second method to improve the diversity of the solutions, alleviate the stagnation and avoid being trapped in local optima. Nazari-Heris et al.^6^, also worked on the optimization of a microgrid, more precisely on the short-term scheduling, taking objectives as cost and emission reduction while considering DRP and uncertainties. The MG comprised an FC and combined heat and power (CHP) units and a battery storage system. They used an ε-constraint method to explain the multi-objective problem while also considering three different scenarios to validate their work. The last scenario had the most promising result: reduced cost and emission. Javidsharif et al.^7^, focused on a not very frequent renewable source: a stream tidal turbine in a microgrid. They used a modified bird mating optimizer (MOBMO) algorithm to solve the multi-objective optimization with mesh-adaptive direct search problem, including cost and emission minimization, while considering storage devices. The microgrid studied is composed of stream tidal turbines, PV, fuel cells, micro-turbines and storage devices. It was observed that an MG consisting of PV and tidal generators is better at delivering load sustainably since they are complementary. Pandey et al.^8^, developed a VPP that comprises a range of RES i.e., solar PV, wind power and FC along with a co-generation unit in a multi-area context. This paper aims to maximize the total profit and simultaneously minimize the generated emission in a single and multi-objective framework. In this work dual scheduling is considered viz., DA scheduling and RT for 24-h and 5-min intervals. The authors proposed an improved version of Harris hawks’ optimization-based metaheuristic technique that can avoid local stagnation and early convergence by ensuring a reduced average computational time to perform the desired optimization. The comparison reveals that the approach is practical as compared to other conventional techniques. Table 1 enlists the approach adopted in the current work and the comparison with the available work performed by researchers. Zhao et al.^9^, studied a pump-supported hydroelectric capacity in a variable market while considering constant water inflows and deterministic power prices. This work uses an optimization algorithm to capitalize on the electrical energy generated and the net profit. The final result is in terms of optimal profit and the optimal energy operation is very similar. Additionally, the price affects the control as the inflow rate increases.

**Table 1:** Comparison of critical literature review and proposed approach.

References	Target objective	Optimization technique	Integration of renewable powered sources, co-gen. unit and reserve provision						Load and electricity price management
			PV	WT	FC	SH	SR	CHP	EP and EM
^1^	Optimized profit and fuel cost	Imbalance mechanism	√	√	X	X	X	√	√
^5^	Reduce operating costs, reduce GHGs	Commercial solver CPLEX	√	X	X	√	√	X	√
^8^	Maximize the total profit and simultaneously minimize the generated emission	Modified Harris hawks’ optimization (MHHO)	√	√	√	X	√	√	√
^10^	Optimization of operating cost	Gravitational search algorithm (GSA)	√	√	√	x	√	X	√
^11^	Cost reduction and optimum sizing of EVCS	Modified teaching–learning-based optimization (TLBO)	√	√	√	X	X	X	X
^12^	Maximize the profit and minimize emission	Multi-objective black widow optimization (MOBWO)	√	√	√	X	X	√	√
^13^	Engineering design problems	Improved GJO	X	X	X	X	X	X	X
^14^	Reduction in operational cost	C & CG	X	√	X	X	X	√	√
^15^	Minimizing operation costs	Relaxed ADMM based optimization	√	√	X	X	√	X	√
This Paper	Reduction of cost and emission	GJO along with BWA and SSA	√	√	√	√	√	√	√

Rehman et al.^16^, listed the existing technologies associated with pumped hydro energy storage (PHES) and the combination of PV and wind turbines i.e., hybrid systems. They emphasized the relevance of PHES as the most suitable technology for massive energy storage, while hybrid systems such as the combination with PV or wind are recommended for island grids followed by a small-scale set-up. Blakers et al.^10^, also explored the potential of PHES for PV and wind turbine-based energy systems. The best storage for those kinds of power plants is pumped hydro and batteries as they balance storage technologies and are suitable for both lengthier and quicker storage periods. Additionally, their cost will be lower for the minimization of the environmental impact and highlighted that off-river PHES is the best solution. Nandini et al.^17^, emphasized the effect of CS load in a DC distribution network by employing various metaheuristic techniques to test operating conditions i.e., power loss index, and reliability followed by voltage stability index. Kushwaha et al.^18^, discussed the challenges regarding the absence of power supply which hinders the development of rural areas in the Indian context. To improve the reliability of power in rural villages, off-grid-based MG is presented and optimized by a slime mould-based optimization algorithm. The presented approach deals with both economic as well as environmental perspectives which comprise solar wind and diesel generators in the MG configuration. Kushwaha et al.^19^, explored socio-technical factors in hybrid renewable-based energy systems by incorporating advanced optimization techniques. The studied system is comprised of biogas and a diesel generator along with batteries and the work is concluded by stating that the Marine predators’ method outperforms the conventional methods namely PSO and GA. Kushwaha et al.^20^, reviewed various aspects of renewable systems in a hybrid framework by considering the configurations, modelling and design parameters to select the most suitable system framework and the installation is as per the requirement of the location i.e., grid-connected or islanded operation. Kushwaha et al.^21^, have extended their study and utilized HOMER software to perform the optimal sizing of renewable-based hybrid systems. PSO method is also utilized in this work through which the cost of energy is reduced which emphasizes both social and technical perspectives. Kushwaha et al.^22^, discussed the cost-effective and environmentally viable dispatch strategy to enhance the reliability of renewable energy systems in a hybrid framework. HOMER software is utilized for result analysis which is supported by SSA and PSO. Kushwaha et al.^23^, discussed load scheduling which can not only reduce the cost associated with power generation but also can increase energy efficiency. HOMER Pro software is utilized to maintain the scheduling by considering customer behaviour and comfort. Kushwaha et al.^12^, presented a load and source side management to improve the performance of renewable systems in a hybrid framework. Marine predator-based optimization is employed to solve the desired problem and it is concluded that the cost of energy is slightly higher. Wu et al.^24^, have presented a regulation model to reduce the additional cost of device integration to enhance the flexibility of integrated energy systems. The increased share of renewables is suggested to reduce the cost further by involving flexible electric and thermal loads to ensure efficient operation. It is also mentioned that the share of renewable should not be excessive, as it reduces the total cost of RES and increases the installed capacity of thermal-based energy storage. The two-stage method of optimization reduces the uncertainty in renewables-based systems and energy storage systems as well.

## Research gap

Based on the thorough review of the available work following are the gaps found in the domain of VPP: One major gap revolves around the absence of a diversity in RERs, viz., small hydro, thermal, or a combination of storage options. These resources are crucial for sustainable energy generation and their absence in the network derails the progress in the domain of VPPs. Besides that, the uncertainties associated with renewable resources and the complementary of some sources were not thoroughly treated in most of the papers while it has a huge impact on the VPPs performance and the optimization of cost and emission. Additionally, the review of the results in some papers indicates that the emission was not significantly affected in the two scenarios with different scheduling. Moreover, integrating ESS, including flexible storage options like PEVs can address numerous risks. By using those technologies, the power indices of the VPP can be improved leading to improved overall performance. The related work reviewed also indicates that none of them included hostile problem formulations that considered simultaneous objectives, i.e., minimizing emissions, and reducing the cost. Also, a small hydro-power resource is also included in the problem formulation, which makes the overall operation of the system more reliable in case of a shortage of power from solar and wind.

### Major contributions

Based on this background, the following are the key contributions performed in this work enlisted as:

- An optimal operation and optimization by the VPP system is discussed with an enhanced share of renewables to reduce the dependence on fossil-based resources.
- The target objectives selected in this work are multi-fold i.e., estimating the minimization of the cost and reduction in the generated emission primarily in a single objective framework which is then extended in a multi-objective context in which simultaneous scheduling is performed in line with a Pareto-based solution.
- A newly established Golden Jackal Optimization is employed to solve the developed problem formulation, and the results are promising in terms of the final output i.e., cost minimization and finally a significant emission reduction.
- Finally, the results are also linked with a few other techniques viz., beluga whale optimization, salp swarm algorithm reported in the literature and the GJO outperforms the other methods in terms of overall performance.

### Paper organization

The rest of the paper is systematized in the following manner. Section “Virtual power plants: the roadmap towards decarbonization” contains the background of the VPP concept and its importance in performing optimal operation and energy management. Section “Problem formulation” reveals the VPP design and the associated resources’ modeling. The selection and relevance of the optimization algorithm for evaluating the selected problem formulation are conferred in Section “Optimization algorithm”. Section “Results and discussion” consists of results and discussion along with a detailed evaluation of the algorithm’s performance for the proposed VPP system with the published papers. Finally, concluding statements are provided in Section “Conclusion” along with possible directions for future work.

## Virtual power plants: the roadmap towards decarbonization

A VPP is a modern approach towards energy management and effective allocation of the DERs. It utilizes digital technology by employing advanced algorithms to integrate multiple decentralized energy resources into a unified and coordinated network to make them work as a single entity. By aggregating various sources such as power procured from solar, wind, BESS, and even electric vehicles which can be utilized as both source and load, a VPP enables efficient monitoring, control, and optimization of power generation and consumption. It performs various functions as a flexible and dynamic power system, capable of responding to grid demands in real-time with minimal human intervention, balancing supply and demand and finally providing ancillary services i.e., voltage balancing, frequency control and congestion management to the electricity grid. A VPP encompasses various components to optimize power generation and management.

**Fig. 1:**
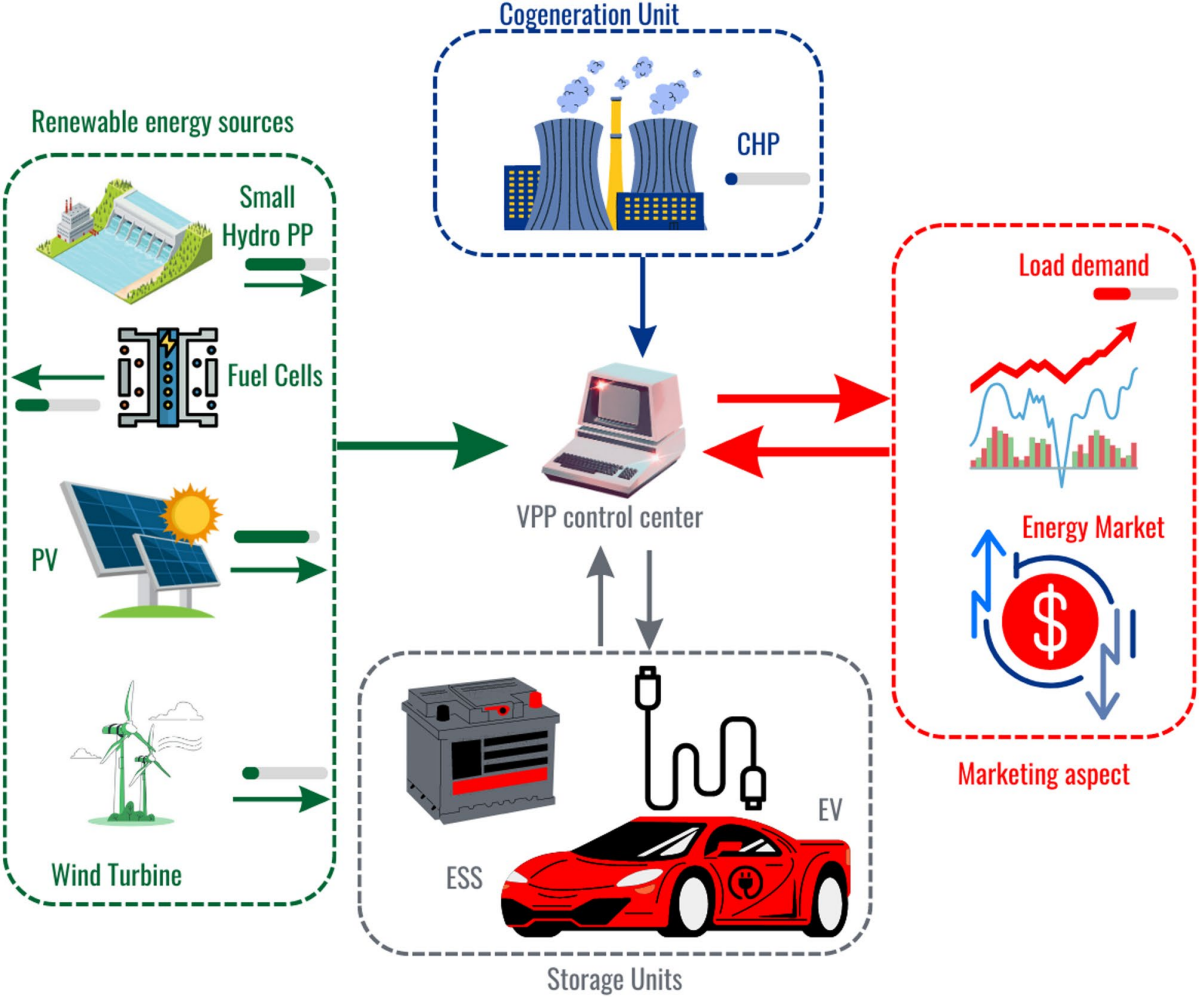
Proposed structure of the VPP system.

Ultimately, it is worth mentioning here that VPP aims to generate profit for its participants by reducing the overall cost of operation involved in the energy-sharing process. VPPs can make prosumers at the center of the energy system by providing energy security round the clock which gives them peace of mind due to the inclusion of power from clean energy sources which also makes them able to contribute to grid-related services. From a safety point of view, VPP reduces the additional strain from the grid by providing them multiple flexible options and a range of curtailable and controllable loads followed by a plethora of non-conventional resources^25^. Employing a VPP that aggregates renewable power generated by solar or wind can be shared or exchanged with the grid during peak loading hours, especially when electricity supplies are strained. The unplanned outages or planned outages termed load shedding can also be reduced by utilizing the services offered by a VPP. The severity and the occurrence of these events can be reduced by advanced cloud-based software that can leverage the power obtained from intermittent renewable sources. Figure 1 contains the structure of the VPP presented in this work in which CHP systems are included in the current configuration to offer the highest efficiency.

**Fig. 2:**
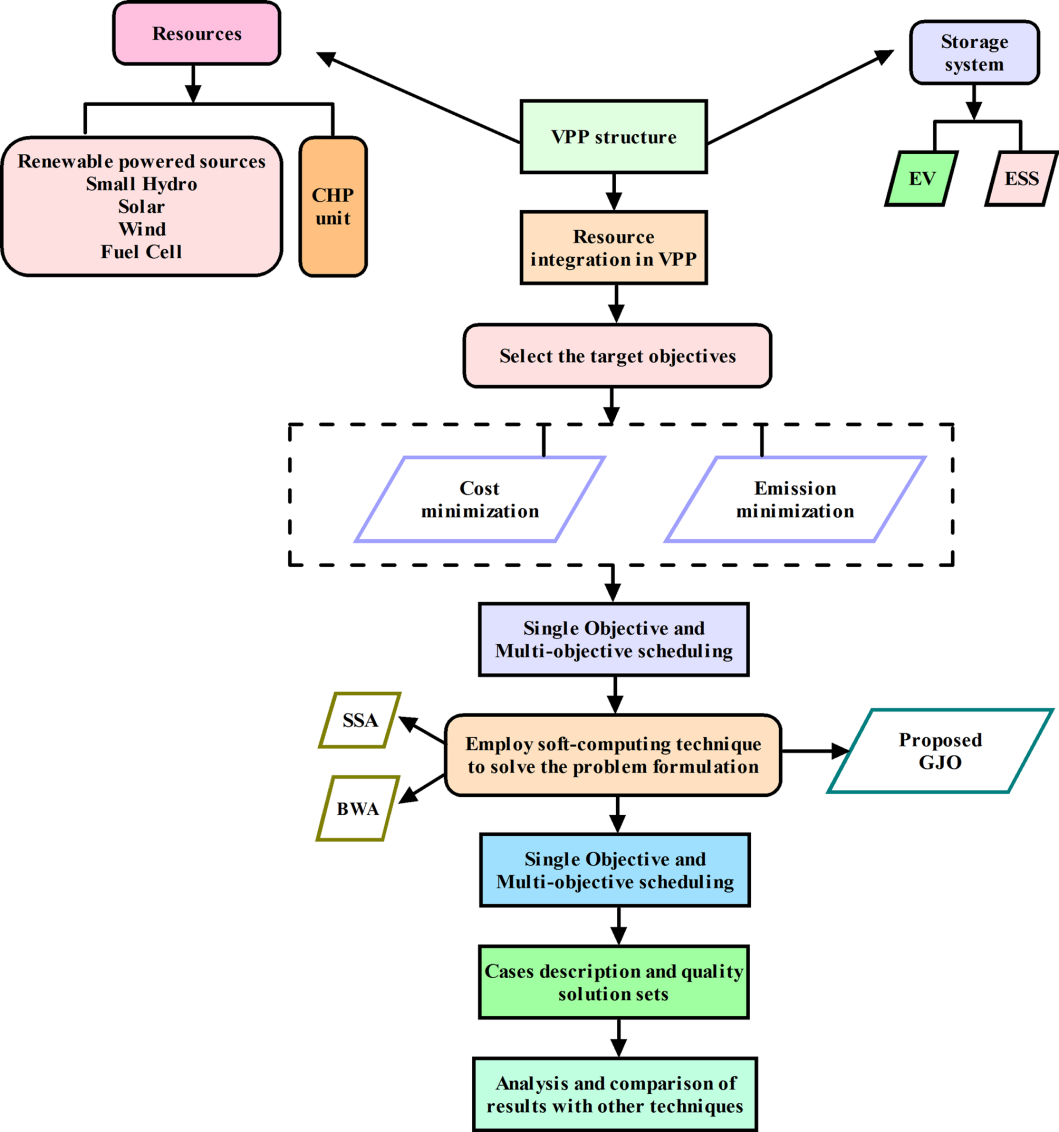
Process flow of the methodological framework.

Multiple renewable energy sources are considered to deepen the renewable portion in the inclusive energy mix i.e., SPV, wind turbines, fuel cells and small hydro (SH). In this work, incorporating renewables is emphasized more to diversify the energy mix to reduce the environmental impact, ultimately promoting sustainable living. Energy storage systems are capable of soaking up the surplus amount of power generated during the day which can be released during peak periods when the cost of electricity is higher. These valuable resources can make the VPP system more robust and make its operation more resilient. By incorporating energy sources, the availability of clean energy generated during day time can be availed as and when required to meet the load demand. This practice also reduces the reliance on traditional coal-based polluting units to balance consumption and generation. The storage provision in the proposed VPP structure is also provided by equipping EVs or PEVs, allowing the VPPs to unleash their storage capabilities and leverage their charging and discharging patterns. In line with EVs, energy storage units (ESUs), such as batteries are also included in the current framework to enable the efficient management and utilization of surplus energy during periods of low demand. The proposed VPP will also be explored in the market and electricity pricing aspect. In fact, two main types of virtual power plants (VPPs) serve different energy management purposes^26^. In Fig. 2, the process flow of the methodological framework is represented in which the target objectives and solving process are mentioned.

## Problem formulation

### Objective function

The target objective function(s) in optimizing and scheduling a VPP in this work aims to optimize multiple objectives. The primary target is to minimize the cost followed by a secondary target to minimize the generated emissions. The objectives are optimized in single objective scheduling which is extended to a multi-objective framework so that simultaneous scheduling can be carried out to optimize the overall cost and emission.

#### Cost minimization

To curtail the total cost, the minimization of costs associated with the operation of energy loss, the purchased energy and the degradation of batteries are considered. The expression of the total cost is Eq. (1). The cost of energy loss is denoted by $C_{energy\_loss}$ which is due to the ohmic loss occurring in the grid lines which is expressed in Eq. (2). The installation cost of the wind and the PV, small hydro, fuel cell and arrays are not considered.

Total cost:

1 $$\begin{gathered} C_{Total\_\cos t} = \sum\limits_{s = 1}^{S} {\pi_{s} } *\sum\limits_{t = 1}^{T} {\left( {C_{Startup\_and\_shutdown\_CHP} + C_{Startup\_and\_shutdown\_SH} } \right.} \hfill \\ \left. {\quad \quad \quad \quad \quad \;\;\;\; + C_{Startup\_and\_shutdown\_FC} + C_{purchased\_energy} + C_{\deg radation\_of\_batteries} } \right) \hfill \\ \end{gathered}$$

where ${\pi }_{S}$ is the probability of scenarios and *s* is a set of scenarios,

*t* is the time ranges from 1 to 24 in Day-ahead scheduling; T is the total time of our simulation which is 24 h.

2 $$C_{energy\_loss} = C_{loss} \sum\limits_{t = 1}^{T} {\left( {\sum\limits_{i = 1}^{N.lines} {\left( {R_{i} *I_{i} (t)^{2} } \right)} } \right)}$$

Operational cost: The cost of acquiring energy from the main grid is $C_{purchased\_energy}$ and the expression is articulated in Eq. (3).

3 $$C_{purchased\_energy} = C_{Grid} \sum\limits_{t = 1}^{T} {E_{Grid} (t)}$$

where $C_{Grid}$ is the cost of buying power from the main grid, and $E_{Grid}$ is the power purchased from the grid.

The cost of battery storage degradation is expressed in Eq. (4).

4 $$C_{degradation\_of\_batteries} = \frac{{C_{investment} *DOD*SOC*\Delta t}}{{N_{CL} }}$$

The expression for depth of discharge is mentioned in Eq. (5).

5 $$DOD = \frac{{SOC_{MAX} - SOC}}{{SOC_{MAX} }}$$

6 $$N_{CL} = \chi *DOD^{\sigma }$$

where *χ* and *σ* signify the parameters of the battery, *SOC* is the energy stockpiled in the battery which is usable energy, ${SOC}_{MAX}$ is the max. energy stored in the battery^14^, *DOD* is the depth of discharge of the battery and ${N}_{CL}$ the life-cycle of the battery.

7 $$C_{Startup\_and\_shutdown\_i} = \alpha_{i}^{t,s} *\left( {1 - \alpha_{i}^{t - 1,s} } \right)*C_{Startup\_i} + \alpha_{i}^{t - 1,s} *\left( {1 - \alpha_{i}^{t,s} } \right)C_{\_shutdown\_i}$$

#### Minimization of emission

Equation (8) expresses another objective function in which emission minimization is carried out as CHP units are involved studied system. It should be noted that the emissions that is considered in this paper are only emissions linked with the grid when the power is procured from the market.

8 $${\text{Emission}} = \min \sum\limits_{{s = 1}}^{S} {\pi _{s} } *\left\{ {\sum\limits_{{t = 1}}^{T} {\sum\limits_{{p = 1}}^{P} {\left( {e_{{pou}} + e_{{hou}} } \right)} } + \left( {e_{{ph}} + e_{{se}} } \right)} \right\}$$

where ${e}_{pou}$ and ${e}_{hou}$ are the emissions by power-only unit and heat-only unit.

From Eqs. (9) and (10) to determine them. Also,$e_{ph}$ and $e_{se}$ are the emission by grid and Eq. (11) is utilized to determine them.

9 $$e_{pou} = \left( {NO_{X}^{pou} + SO_{2}^{pou} + CO_{2}^{pou} } \right)*P_{t,p}^{pou}$$

10 $$e_{hou} = \left( {NO_{X}^{hou} + SO_{2}^{hou} + CO_{2}^{hou} } \right)*P_{t,p}^{hou}$$

11 $$e_{ph} + e_{se} = \left( {NO_{X}^{ph + se} + SO_{2}^{ph + se} + CO_{2}^{ph + se} } \right)*P_{t,p}^{ph + se}$$

#### Framework of multi-objective problem

Researchers apply Heuristic optimization techniques to deal with multi-objective problems, and the results are promising^15,27^. To obtain the most likely solution between the conflicting objectives, the problem formulation for VPP is handled by a weighting factor method expressed in (12) in a dual-objective outline. Identical weightage is allocated to the target objective to ensure symmetry among the final output.

12 $${\text{Fitness}} = w1 \times {\text{Total\_Cost}} + w2 \times {\text{Emission}}$$

### Resources in the proposed VPP system

#### Power generated by a small hydro power

It is one of the oldest sources of RE but generally does not count fully as one of the non-conventional sources. Recently small-hydro are categorized as a renewable source which is the main motive for including small hydro power (SHP) in the network of DERs. Hydro power is generated from the force of the natural flow of water. Hydro power technologies harness the power of water by utilizing the vertical drop established by a dam or diversion structure, between the incoming flow on one side and the outgoing flow far below on the other, to generate electricity. In the developed VPP, SHP makes a huge impact in maintaining power balance in the grid by quickly adjusting the power generated and responding rapidly as and when solar and wind are unable to fulfill the demand. Also, hydro’s uncertainty aspect is minimal compared to the inherently varying nature of wind and solar. In India, hydro power plants with a size of 25 MW or below are considered small hydro. Equation (13) expresses the mathematical expression associated.

13 $$P_{SH}^{s,t} (G^{t} ) = G^{t} *g*H*\eta$$

where $P_{SH}^{t}$ expresses the power generated by a small hydro unit, ${G}^{t}$ is the mass flow rate at an interval *t* for scenario *s*, *g* Gravitational constant and *H* is the net head, which represents the physical head for accounting for the losses, $\eta$ is the total efficiency which is the product of the efficiency of the turbine, drive system and generator.

#### Solar photovoltaic

To study the power of the PV modules we have to know the characteristics of the modules as well as the solar irradiation in the specific chosen place as well as the ambient temperature. The output power of the solar photovoltaics ${P}_{PV}^{s,t,p}$ is given in Eq. (14)^8^.

14 $$P_{PV}^{s,t} \left( {Ir^{s,t} } \right) = V^{s,t} *I^{s,t} *FF*N_{{\text{modules}}}$$

15 $$FF = \frac{{I_{MPP} *V_{MPP} }}{{I_{SC} *V_{OC} }}$$

16 $$V^{s,t} = V_{OC} - K_{V} *T_{C}^{s,t}$$

17 $$I^{s,t} = Ir^{s,t} *\left[ {I_{SC} + K_{I} *(T_{C}^{s,t} - 25)} \right]$$

where FF is the Fill Factor, ${I}_{MPP}$ and ${V}_{MPP}$ are the max. power point current and voltage, ${N}_{modules}$ is the number of modules, ${Ir}^{s,t}$ is solar radiation, ${V}_{OC}$ and ${I}_{SC}$ is open circuit voltage and short-circuit current, ${K}_{I}$ and ${K}_{V}$ denotes respectively the current and the voltage temperature coefficients, ${T}_{C}^{s,t}$ is the temperature of a solar cell.

#### Wind power

Wind is the most extensively used resource categorized as a potential non-conventional energy source. The output power generated from wind turbines $P_{WT}^{s,t}$ is calculated using (18) and the modeling is adapted from^28^.

18 $$P_{{WT}}^{{s,t}} = \left\{ {\left. \begin{gathered} N_{{WT}} *P_{{rated}} *\left( {\frac{{V^{t} - V_{b} }}{{V_{e} - V_{b} }}} \right)^{3} ;\quad \;\,V_{b} \le V^{t} < V_{e} \hfill \\ N_{{WT}} *P_{{rated}} ;\quad \quad \quad \quad \quad \quad \quad \quad V_{b} \ge V^{t} ,V_{S} < V^{t} \hfill \\ P_{{Wind}} ;\quad \quad \quad \quad \quad \quad \quad \quad \quad \quad \quad V_{b} \ge V^{t} ,V_{S} < V^{t} \hfill \\ \end{gathered} \right\}} \right.$$

where ${\text{P}}^{wind}$ is output power generation, ${\text{P}}^{rated}$ denotes rated power, and $V_{t}^{b}$ is the wind speed. $V^{b}$, $V^{s}$ is cut-in and cut-out speed followed by $V^{e}$ which is rated wind speed.

#### Fuel cells unit

The output power of the FC unit $P_{FC}^{t}$ at *t*th interval and is the function of PLR. The concept is adapted from ^8^ and the expressions to determine are depicted in Eq. (19).

19 $$P_{FC}^{t} = \left\{ {\left. \begin{gathered} \eta_{FC}^{t} = 2.2716;r_{FC}^{t} = 6.6816 \hfill \\ PLR_{t} < 0.05; \hfill \\ \eta_{FC}^{t} = 0.9033*PLR_{t}^{5} - 2.9996*PLR_{t}^{4} + 3.6503*PLR_{t}^{3} - 2.0704*PLR_{t}^{2} + 0.4623*PLR_{t}^{1} + 0.37 \hfill \\ PLR_{t} \ge 0.05; \hfill \\ r_{FC}^{t} = 1.0785*PLR_{t}^{4} - \, 1.9739*PLR_{t}^{3} + \, 1.5005*PLR_{t}^{2} - 0.2817*PLR_{t}^{1} + \, 0.6838 \hfill \\ \end{gathered} \right\}} \right.$$

where PLR is part load ratio, $\eta_{FC}^{t}$ is the efficiency of FC and $r_{FC}^{t}$ is the heat-to-electricity ratio FC.

The power extracted by the ${P}_{FC}^{t}$ unit is constrained to go beyond the generated power in the earlier interval ${P}_{FC}^{t-1}$ greater than a certain amount which is the ramp rate boundary of FC.

20 $$\Delta P_{FC\_down} .T \le P^{t}_{FC} - P^{t - 1}_{FC} \le \Delta P_{FC\_up} .T$$

21 $$P_{FC\_\min } \le P^{t}_{FC} \le P_{FC\_\max }$$

where $\Delta P_{FC\_down} .T$ is down ramp limit and $\Delta P_{FC\_up} .T$ is FC up ramp limit, $P^{t}_{FC}$ is power produced by FC at the current interval and $P^{t - 1}_{FC}$ is power generated by FC at the previous interval.

#### Power switching between grid and CHP unit

Two types of CHP units are considered and the constraint of output power is represented in (22) and (23).

22 $$0 \le P_{t,p}^{chp} \le P_{p,A}^{chp} \times V_{t,p}^{chp}$$

23 $$0 \le H_{t,p}^{chp} \le p_{p,B}^{chp} \times V_{t,p}^{chp}$$

where $P_{t,p}^{chp}$ is the electric power output of CHP and $V_{t,p}^{chp}$ is commitment status of CHP.

#### Reserve option

##### Battery-based storage system

BESS are also utilized to maintain stability due to their shorter response time span which can be utilized in the events of scarcity from renewable sources. The modelling is represented in Eq. (24):

24 $$P_{ESS}^{i} = \sum\nolimits_{t} {\sum\nolimits_{s} h } C_{S,\deg }^{s} \left( {P_{S,ch}^{s,t} + P_{S,dch}^{s,t} } \right)$$

where $C_{S,\deg }^{s}$ is the cost of ESS deprivation and $\left( {P_{S,ch}^{s,t} + P_{S,dch}^{s,t} } \right)$ is charging/ discharging of ESSs.

The SoC is assessed as per (25) and the charging and discharging is restricted as per (26, 27). Interested readers can explore the detailed modelling explained in^8^.

25 $$E_{S}^{s,t} = E_{S}^{s,t - 1} + \left[ {\left( {\eta S,chP_{S,ch}^{s,t} } \right) - \left( {\frac{1}{{\eta_{S,dch} }}P_{S,ch}^{s,t} } \right)} \right]$$

26 $$0 \le \left( {\eta_{ESS,ch}^{e} P_{ESS,ch}^{s,t} } \right) \le u_{ESS,ch}^{s,t} P_{ESS,ch,\max }^{e}$$

27 $$0 \le \left( {\frac{1}{{\eta_{ESS,dch}^{e} }}P_{ESS,dch}^{s,t} } \right) \le u_{ESS,dch}^{s,t} P_{ESS,dch,\max }^{e}$$

where $\eta_{ESS,ch}^{e}$ and $\eta_{ESS,dch}$ is charge and discharging efficiency of ESSs. $P_{ESS,ch,\max }^{e}$ and $P_{ESS,dch,\max }^{s}$ are max. charge and discharge rate of ESS.

##### Electrical vehicles as secondary reserves

By enabling EVs as a reserve option, the participants involved in VPP can be profited as vehicle owners by earning some additional incentives and the VPP operator has additional power options during any shortage of power. The formulation is mentioned in the following equations.

28 $$P_{EV}^{i} = \sum\nolimits_{t} {\sum\nolimits_{e} h } \left( {\lambda_{price}^{w,t} P_{EV,ch}^{e,t} + C_{EV,dis}^{w,t} P_{EV,dch}^{e,t} } \right)$$

29 $$C_{EV,dch}^{w,t} = FEV,dch\lambda_{price}^{w,t}$$

where $P_{EV,ch}^{e,t}$ and $P_{EV,dch}^{e,t}$ is charging and discharging of EV and $\lambda_{price}^{w,t}$ is the electricity price,$C_{EV,dis}^{w,t}$ is the cost of EV. EVs charging and discharging rate in (30, 31) and further details regarding the formulation are available in ^8^.

30 $$0 \le \left( {\eta_{EV,ch}^{e} P_{EV,ch}^{e,t} } \right) \le u_{EV,ch}^{e,t} P_{EV,ch,\max }^{e}$$

31 $$0 \le \left( {\frac{1}{{\eta_{EV,dch}^{e} }}P_{EV,dch}^{e} } \right) \le u_{EV,dch}^{e,t} P_{EV,dch}^{e,\max }$$

where $\eta_{EV,ch}^{e}$ and $\eta_{EV,dch}^{e}$ is charge and discharge efficiency. $P_{EV,ch}^{e,\max }$ and $P_{EV,dch}^{e,\max }$ are max. charge and discharge of EV.

### Power balance in the network

The electrical power balance of the system is maintained during the operation as depicted in Eq. (32). The equivalent output power at an interval *t* is denoted as $P_{eqv}^{t,p}$. The power balance equation comprises all of the resources associated with the proposed VPP system.

32 $$P_{eqv}^{t,p} = a_{s,t} *P_{WT}^{t} + b_{s,t} *P_{PV}^{t} + c_{s,t} *P_{SH}^{t} + d_{s,t} *P_{FC}^{t} + e_{s,t} *P_{CHP}^{t} + f_{s,t} *P_{EV}^{t} + g_{s,t} *P_{ESS}^{t} - P_{Load}^{t}$$

where ${P}_{CHP}^{t}$: Power exchange between the grid and the CHP at an interval *t*, ${P}_{WT}^{t}$: Power produced by the wind turbine at an interval *t*, ${P}_{PV}^{t}$: Power produced by the solar photovoltaic at an interval *t*, ${P}_{SH}^{t}$: Power generated by a small hydro at an interval *t*, ${P}_{FC}^{t}$: Power generated by fuel cell at an interval *t*, ${P}_{EV}^{t}$: Power available from electric vehicles as storage at an interval *t*, ${P}_{ESS}^{t}:$ Power available from ESS as storage at an interval *t*, ${P}_{Load}^{t}:$ Represents the electric loads at an interval *t*, ${a}_{s,t}$…${g}_{s,t}$: Relative coefficient shares of every resource in the scenario s at interval *t*.

## Optimization algorithm

“Metaheuristic algorithms” are optimization approaches in which the algorithm performs a local and global search for the given problem space through an iterative process to obtain an optimum or near-optimal solution. It is mainly divided into a few categories: single-point search, population-based method and evolutionary search mechanism. Mixed integer linear programming (MILP) and linear programming (LP) are employed to solve problems that are not restricted to any particular domain. LP only deals with continuous variables while MILP involves both continuous and integer variables as well and therefore, the MILP method is known for its accuracy. However, this approach is relatively conventional as the desired results are optimal but the computational time is more. Many optimization techniques have been developed in the past few years ranging from nature-inspired, bio-inspired and so on. Considering the nature of the problem, it is suitably decided which method is more relevant and efficient ensuring their solving capability. Therefore, advanced optimization techniques are generally preferred to solve engineering problems. Some widely explored and established examples of optimization techniques are genetic algorithm (GA), differential evolution (DE), ant colony optimization (ACO), PSO, bacterial foraging optimization (BFO), artificial bee colony (ABC), SSA and BWA to name a few. In this work, Golden Jaclal optimization is selected to solve the cost/emission problem of the VPP system. A detailed description of GJO is discussed in the subsequent section.

### Golden Jackal optimization

**Fig. 3:**
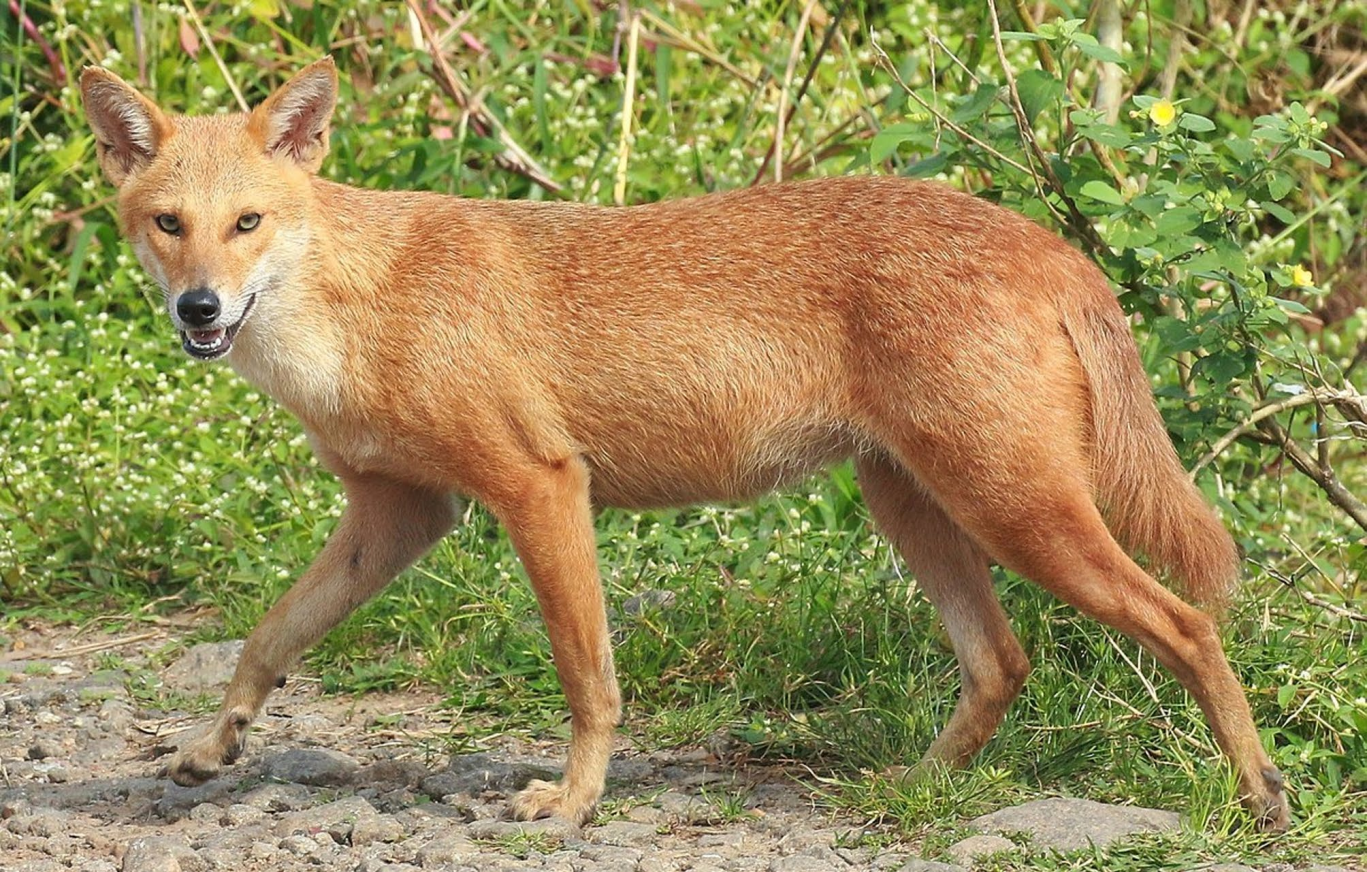
Golden Jackal (*Canis aureus*)^29^.

The Golden Jackal-based optimization mimics the collaborative hunting behavior of golden jackal which imitates biological swarm intelligence. The hunting relies on three phases i.e., noticing, encircling and stimulating, finally attacking the prey. Figure 3 shows Jackal in the wild^29^.

#### Search space model

In the primary phase, the random location of the prey is well-defined as a matrix with *N* rows representing the number of populations and *n* columns referring to the dimensions. At first, the solutions are spread uniformly over the search space and the formulation for the same can be expressed in (33).

33 $$Y_{0} = Y_{\min } + rand\left( {Y_{\max } - Y_{\min } } \right)$$

where *rand* is a random type vector in the (0 to 1) range, $Y_{\max }$ and $Y_{\min }$ are the upper and lower bounds.

The initialization results in a matrix of *prey* in which the jackal pair forms based on their individual fitness.

34 $${\text{Prey}} = \left[ {\begin{array}{*{20}c} {Y_{1,1} } & {Y_{1,2} } & {...} & {Y_{1,d} } \\ {Y_{2,1} } & {Y_{2,2} } & {...} & {Y_{2,d} } \\ \begin{gathered} Y_{3,1} \hfill \\ \vdots \hfill \\ \end{gathered} & \begin{gathered} Y_{3,2} \hfill \\ \vdots \hfill \\ \end{gathered} & \begin{gathered} ... \hfill \\ \vdots \hfill \\ \end{gathered} & \begin{gathered} Y_{3,d} \hfill \\ \vdots \hfill \\ \end{gathered} \\ {Y_{n,1} } & {Y_{n,2} } & {...} & {Y_{n,d} } \\ \end{array} } \right]$$

A fitness i.e., objective function is functional to evaluate the fitness of prey individually during the optimization and the following matrix accumulates the fitness of all prey.

35 $$F_{OBJ} = \left[ {\begin{array}{*{20}c} {f(Y_{1,1} } & {Y_{1,2} } & {...} & {Y_{1,d} )} \\ {f(Y_{2,1} } & {Y_{2,2} } & {...} & {Y_{2,d} )} \\ \begin{gathered} f(Y_{3,1} \hfill \\ \vdots \hfill \\ \end{gathered} & \begin{gathered} Y_{3,2} \hfill \\ \vdots \hfill \\ \end{gathered} & \begin{gathered} \cdots \hfill \\ \vdots \hfill \\ \end{gathered} & \begin{gathered} Y_{3,d} \hfill \\ \vdots \hfill \\ \end{gathered} \\ {f(Y_{n,1} } & {Y_{n,2} } & {...} & {Y_{n,d} } \\ \end{array} } \right]$$

where *n* denotes the prey and *d* refers to the variable and $F_{OBJ}$ is matrix to accumulate the fitness of prey, *f* is the objective function.

#### Exploration phase

The strategy used by the jackal is discussed in the phase. Generally, the natural tactics are applied by jackals to perform the hunt, but due to the evading energy of prey, it is at times difficult to convert it into a successful hunt. Therefore, the search agent viz., jackals remain still and search for other potential prey which a male and female jackal lead follows it, respectively.

36 $$Y_{1} (t) = Y_{M} (t) - E.\left| {Y_{MJ} (t) - rl.{\text{Prey}}(t)} \right|$$

37 $$Y_{2} (t) = Y_{FM} (t) - E.\left| {Y_{FMJ} (t) - rl.{\text{Prey}}(t)} \right|$$

where *t* is the current iteration and ${\text{Prey}}(t)$ denotes the position type vector of prey. $Y_{1} (t)$ and $Y_{2} (t)$ are the updated position of the Jackal corresponding to the prey. $Y_{MJ}$ and $Y_{FMJ}$ is the location of male and female Jackal and $rl$ is a random vector as per levy distribution.

38 $$E = E_{1} \times E_{0}$$

where *E* is the evading energy of prey, *E*_0_ is initial energy and *E*_1_ is the deteriorating energy of prey.

The initial energy *E*_0_ deviates randomly between (-1 to 1). The prey weakens substantially when this value further declines from 0 to (-1) and on the contrary when this value rises from 0 to 1, the prey regains their strength and cannot be captured by the Jackals.

39 $$E_{0} = 2 \times r - 1$$

where *r* is an arbitrary number in the range of 0 and 1.

It is worth mentioning here that *E*_*1*_ is decreased from 1.5 to 0 as the iteration progresses. Also, the distance between Jackal and the target is computed by $\left| {Y(t) - rl.{\text{Prey}}(t)} \right|$. The distance is computed for Jackal’s present location as per the prey’s evading energy.

40 $$E_{1} = c_{1} \times (1 - (t/T))$$

where *c*_1_ is a constant value assigned to 1.5 and *T* denotes the maximum number of iterations.

41 $$rl = 0.05 \times LVF(y)$$

where *LVF* levy fight function.

42 $$LVF(y) = 0.01 \times (\mu \times \sigma )/\left( {\left| {v^{(1/\beta } } \right|} \right)$$

43 $$\sigma = \left( {\frac{{r(1 + \beta ) \times \sin \left( {\tfrac{\pi \beta }{2}} \right)}}{{r\left( {\tfrac{1 + \beta }{2}} \right) \times \beta \times 2^{{\left( {\frac{\beta - 1}{2}} \right)}} }}} \right)^{{\frac{1}{\beta }}}$$

where *u* and *v* are random num. in the range of 0 and 1. *β* is the default constant equivalent to 1.5.

**Fig. 4:**
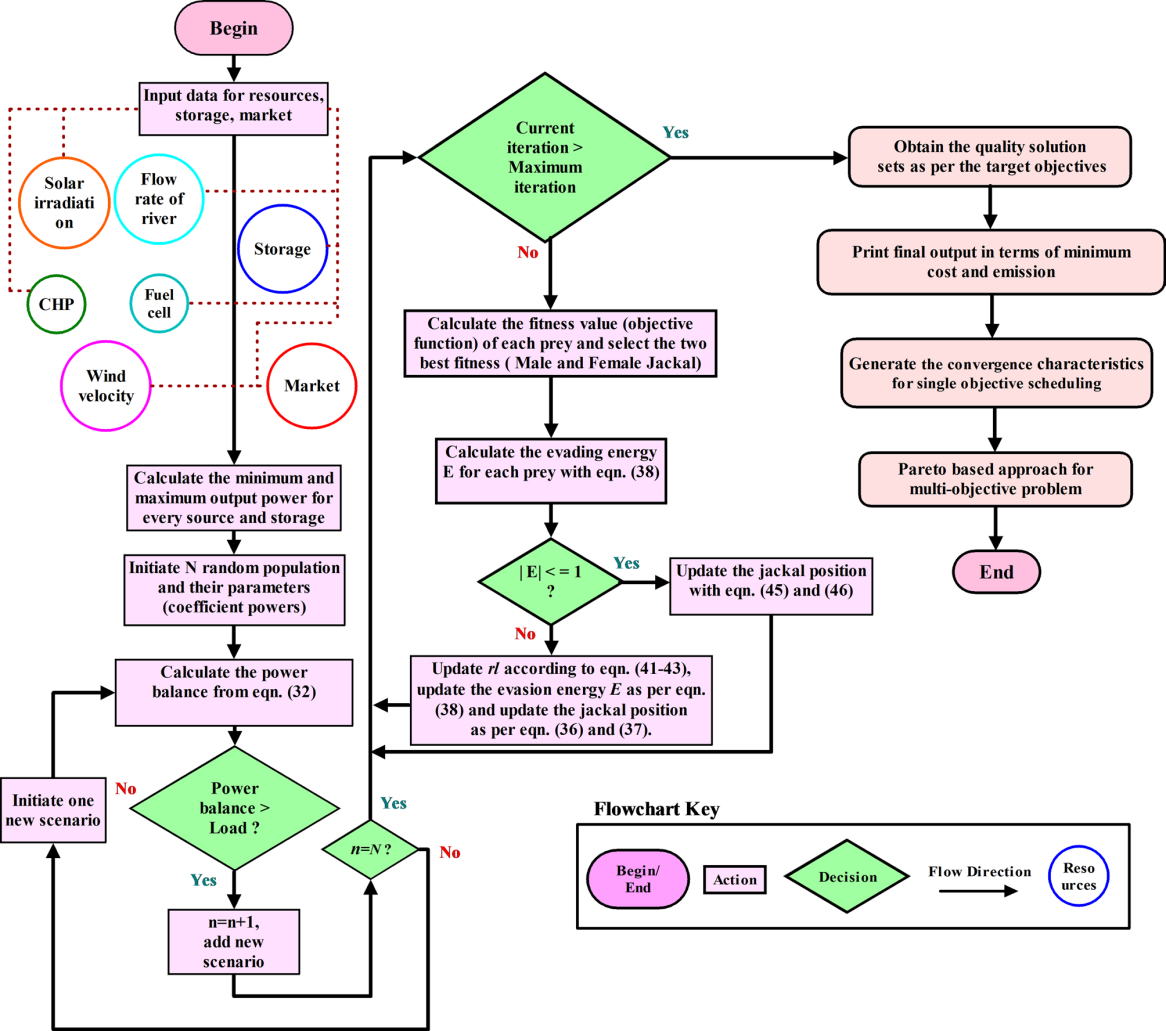
Flowchart for GJO optimization.

Therefore, by taking the average of (36) and (37), the positions of Jackals are updated. A detailed flowchart for the selected optimization is depicted in Fig. 4.

44 $$Y(t + 1) = \frac{{Y_{1} (t) + Y_{2} (t)}}{2}$$

#### Exploitation phase

After the exploration phase, enclosing and pouncing are performed by the jackals. When the prey is beleaguered by jackals its escaping energy starts depleting and the best jackal pair encloses the prey spotted in the previous state. After enclosing, they swoop on prey and demolish it. The formulation is represented in (45) when both male and female Jackals hunt together.

45 $$Y_{1} (t) = Y_{M} (t) - E.\left| {rl.Y_{MJ} (t) - {\text{Prey}}(t)} \right|$$

46 $$Y_{2} (t) = Y_{FM} (t) - E.\left| {rl.Y_{FMJ} (t) - {\text{Prey}}(t)} \right|$$

where $Y_{1} (t)$ and $Y_{2} (t)$ are the updated position of Jackal, $Y_{FMJ} (t)$ and $Y_{MJ} (t)$ are the location of female and male Jackal.

The function $rl$ in (45) and (46) is included in the formulation to bypass the local optima stagnation and enhance the exploration capability. Including this function plays a vital role during the final phase of optimization as the stopping criteria are set once the desired number of iterations is reached.

#### Transition phase from exploration to exploitation

Thus, the process begins with generating a random population considered potential solutions. During the iteration course, the pair of Jackals should determine the prey’s anticipated position. The *E*_*1*_ parameter is abridged from 1.5 to 0 for underlying exploration and exploitation. The hunting pairs of Golden Jackal diverge from prey when *E* > 1 and perform a successful hunt when prey energy is *E* < 1. Finally, the algorithm is concluded when the stopping criteria are satisfied and the best solution is printed in terms of cost and emission. The employed GJO technique performs well in similar real-world engineering problems and achieves multitask optimization problems in less computational time. It has good robustness and quickly converges in each case.

The GJO optimization technique is utilized to solve the VPP problem due to its characteristics of maintaining the balance between exploration and exploitation. This particular feature is regarded as the most important for any optimization method and the selection has been made by considering its ability and performance for the benchmark functions. Also, the parameters related to GJO are provided to have more clarity from the reader’s perspective in Table 2. Moreover, a detailed discussion is also included regarding the specific outputs i.e., minimum cost and emission, maximum cost and emission and also the computational time required by GJO to reach the near-global optimum solution. The selected technique is also compared with other established methods BWA and SSA and upon comparison, it is observed that GJO outperforms these techniques and ensures the robustness of the algorithm.

**Table 2:** Algorithm-specific parametric settings.

Optimization techniques	Algorithm specific parameters		
GJO	Constant c_1_ = 1.5	Arbitrary number r = 0 to 1	Initial energy level E_0_ = -1 to 1
BWA	Constant β = 1.5	Random number B_0_ = 0–1	Exploration phase B_f_ > 0.5 Exploitation phase B_f_ ≤ 0.5
SSA	Random variables r_1_, r_2_, r_3_ = [0, 1]	Coefficient c = 0.5	Initial speed v_0_ = 0
Population size and Iterations (Max. number) for all the employed techniques = 20 and 200			
Number of independent runs = 100			

Many advantages of GJO are discussed such as its simple structure with minimal control parameters which makes the approach relatively less complex as compared to other techniques. The exploration ability of GJO is also remarkable which results in improved convergence trend at a faster rate without utilizing many iterations. The ability of the solution refinement in the promising and potential regions is also a plus point of this optimization. Each optimization techniques have some limitations and while selecting any specific technique as a solver the limitations should be in consideration. The employed technique GJO has the following limitations: the algorithm struggles to reach an optimum solution when applied for search spaces with higher dimensions. However, incorporating adaptive parameter tuning and modified initialization strategies can reduce this limitation to a certain extent. The analysis performed in this work is duly validated with the relevant papers reported in the literature. As there are limited studies available in the VPP domain, the most relevant ones are selected and co-related with the proposed approach. Upon comparison with the published papers, it is observed that the presented method performs well not in in terms of quality solutions obtained from the selected optimization technique GJO but also in terms of improved efficiency i.e., reaching the desired solution in a minimum number of epochs. Also, the computational complexity is relatively reduced which improves the overall performance and therefore, the explored method is more viable in terms of practicability.

### Additional techniques

#### Beluga whale optimization algorithm

Beluga whale algorithm-based optimization is another swarm-based method utilized for solving various optimization-based problems. It is inspired by the activities of beluga whales (BWs), which comprise of multiple phases i.e., exploration and exploitation phase followed by whale fall corresponding to the behaviors of pair swim, prey, and whale fall, respectively. In the exploration phase, the pair usually swims with two beluga whales closely together in a harmonized manner. Thus, the locations of search agents are determined by this pair swim of BWs^30^. In the exploitation phase, BWs share the location information of prey to consider the best candidate among the others. BWA supposes that it can clasp the prey by performing a levy flight approach.

#### Modified version of salp swarm algorithm

The salp swarm-based algorithm^31^ is enthused by the method of probing food that salps exhibit as a swarm behavior which results in the formation of a salp-chain. The population in a salp chain involves a leader and a cluster of followers in which the leader pursues a food source and the followers alter their location with respect to the salp eventually reaching towards the leader. SSA can be framed in a single and multi-objective approach by utilizing a fixed-sized collection that retains the best results until the iterative process reaches a stopping condition. An improved method is offered during the search process which is directed mostly by the leader among the salp, which may lead to an underwhelming performance as the leader is the only self-governing agent within the population in the original version of SSA. Therefore, authors in^32^ introduced a new category of search agents and termed it as exploring salps.

## Results and discussion

The target objective function considers multiple objectives to carry out the intended optimal scheduling of the developed VPP consisting of a diverse range of DERs, including PV, wind power, FC units, and small hydro (SH), which are merged to form a holistic VPP. Along with renewables a cogeneration unit is also equipped with the current configuration system under consideration in line with multiple storage options in the arrangement of EV as flexible reserve and ESS applicable as spinning reserve. The study is performed in a MATLAB environment having the installed version of MATLAB 2022b with 16 GB of random-access memory in 11th Gen Intel® Core i7™ processor clocking at a speed of 3.30 GHz.

### Numerical analysis

**Fig. 5:**
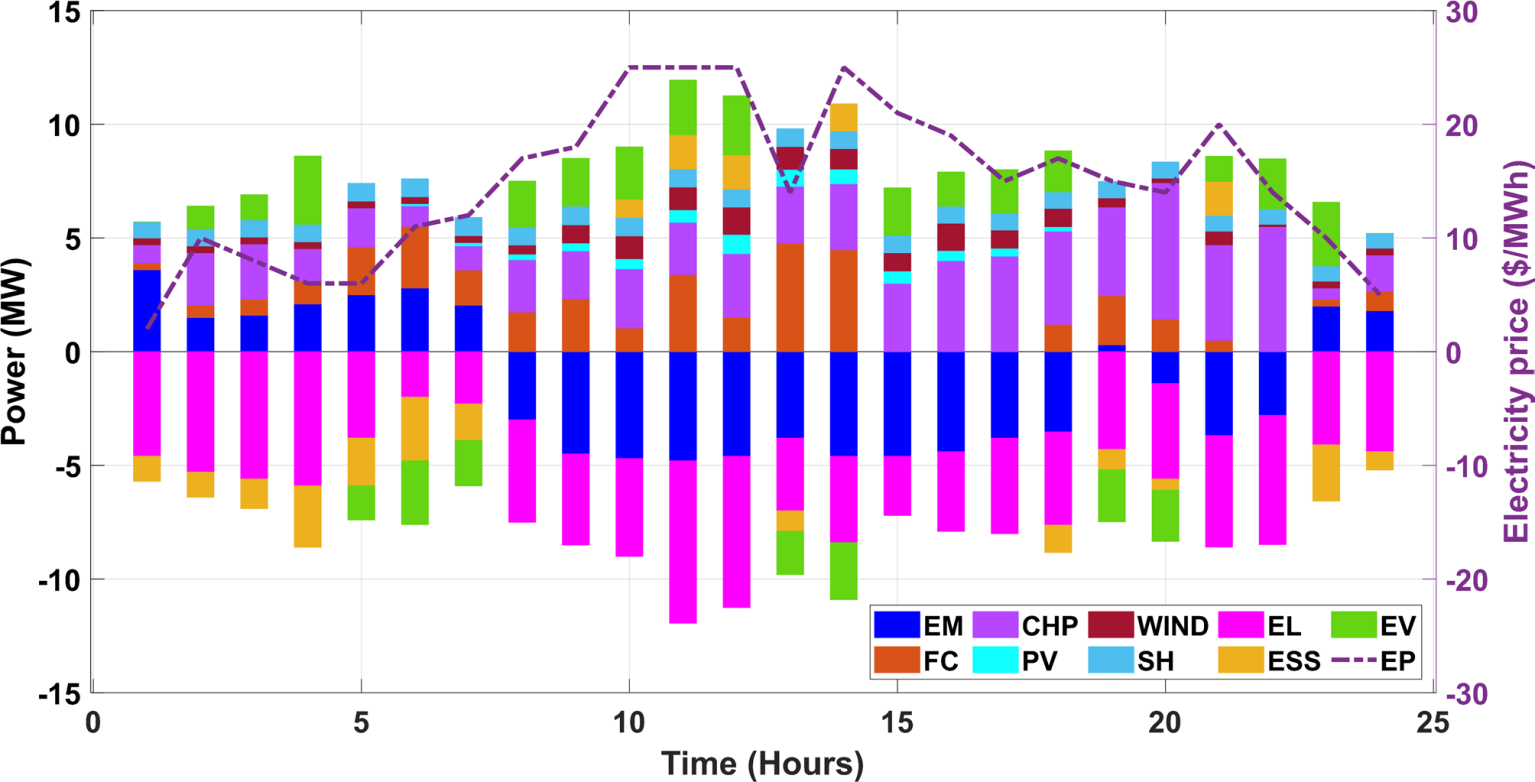
Power balancing for Case I in VPP network.

**Fig. 6:**
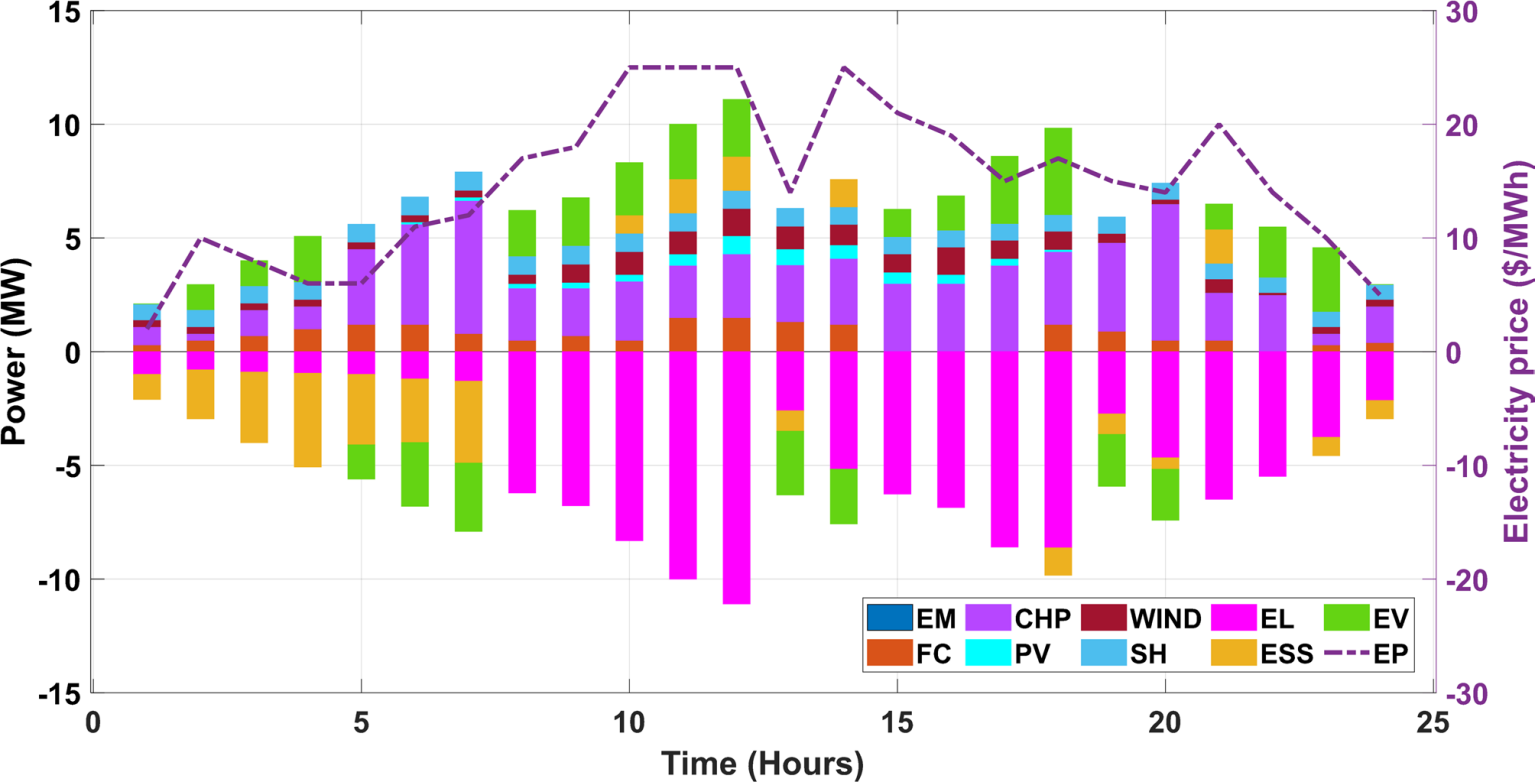
Power balancing for Case II in VPP network.

The proposed formulation consists of a single as well as multi-objective framework in which the primary objective is to minimize the cost associated with the system. In addition, the emission aspect is also given due consideration in this work as the CHP unit is selected as a resource that can provide decent efficiency but also emit a certain amount of emission. To curb this generated emission, a reduction of the same is also carried out in the proposed work. All the objectives are initially solved in a single objective framework which is then extended to a multi-objective framework due to their conflicting nature. Therefore, a Pareto-based approach is utilized, giving the VPP operative a chance to select the best one as per the requirement. It is worth mentioning here that due to the non-linear characteristics of the selected problem, a recently developed advanced optimizer is employed in this work i.e., GJO which performs better in finding the global solution. For demonstration purposes, the obtained results are also linked with other techniques, revealing the proposed formulation’s efficacy. Also, the data pertaining to solar and wind are taken from^33^ and the rest of the data are referred from^8^. The power balance for the day-ahead scheduling is offered in Figs. 5 and 6 for Case I and Case II. Also, the energy market and electricity price are applicable in this work since the market infrastructure is a crucial aspect of any VPP system. The bar graphs are assigned different colours to enhance the clarity among various resources. Also, the abbreviation for each source are as follows, energy market is (EM), solar photovoltaic is denoted as (PV), fuel cell unit are (FC), small hydro is (SH). Also, energy storage system is (ESS) and electric vehicle is denoted by (EV) followed by electricity price (EP). The renewables are considered in the system configuration to enhance the share of non-conventional energy sources (NCERs) in the energy mix to reduce the reliance on fossil-based energy generation resources. The inherent nature of uncertainties makes renewable integration challenging and therefore share from the renewables is relatively lower as compared to conventional-based sources. The accurate prediction and forecast of the renewables play utmost importance which is possible by employing advanced techniques like ARIMA, RBFNN, SARIMA and ANFSIS to name a few^34^. The analysis performed in this work is validated in our previous work and the uncertainties aspect is also explored^35^. Considering the previous analysis, another important renewable source is included in the energy mix i.e., small hydro (SH) to cope with the complexities and uncertainties of weather-dependent resources viz., solar and wind in the present study.

### Description of cases

Multiple cases are discussed explicitly in which Case I deals with cost reduction followed by Case II, which is the minimization of emissions in a single objective framework. In addition, the target objectives are solved in Case III simultaneously in which cost and emission are reduced and the analysis with different techniques is performed.

#### Case I: single objective scheduling (minimization of cost)

In this case, an attempt is made to minimize the associated cost for the developed system. To carry out the optimal operation of the developed VPP network, renewable-based power generating resources are selected which are flexible and can be adjusted to fulfil the power requirement as per the need. During the day time when the sun is shining, solar power can deliver the power along with wind resources when the flow of wind is in full swing.

A fuel cell unit is also included, which can fulfil the demand as per its capability. In addition, small hydro is selected as one of the resources in the current configuration of VPP under study as they are also considered renewable power sources. The costs associated with these sources are duly considered and to improve the overall working and responsiveness of the system, costs are minimized. The numerical values are enlisted in Table 3 and the parameters are minimum cost, maximum cost followed by mean cost. The quality sets of solutions are printed after multiple independent runs so that the optimum solutions’ quality is retained without compromise. Furthermore, the attained results are also compared with the reported work aligning with the proposed formulation which can be observed in Table 3. The percentage reduction in the obtained cost from the proposed GJO is 5.84% when compared with DRACCF and 10.73% when linked with the column and constraint gradient (C&CG) method. Also, the selected technique in this work performs better when compared with other techniques i.e., SSA and BWA by 2.1% and 0.62%, respectively.

**Table 3:** Performance assessment with employed techniques for Case I.

Output ($)	Proposed GJO	BWA	SSA	C & CG^36^	DRAACF^37^
minimum cost	5712.71	5748.42	5835.6	6400	6066.46
Maximum cost	7185.23	7224.36	7298.3	-	-
Mean cost	6448.97	6486.39	6566.95	-	-
Computational run time (sec.)	181.97	235.21	261.46	-	342.87

**Fig. 7:**
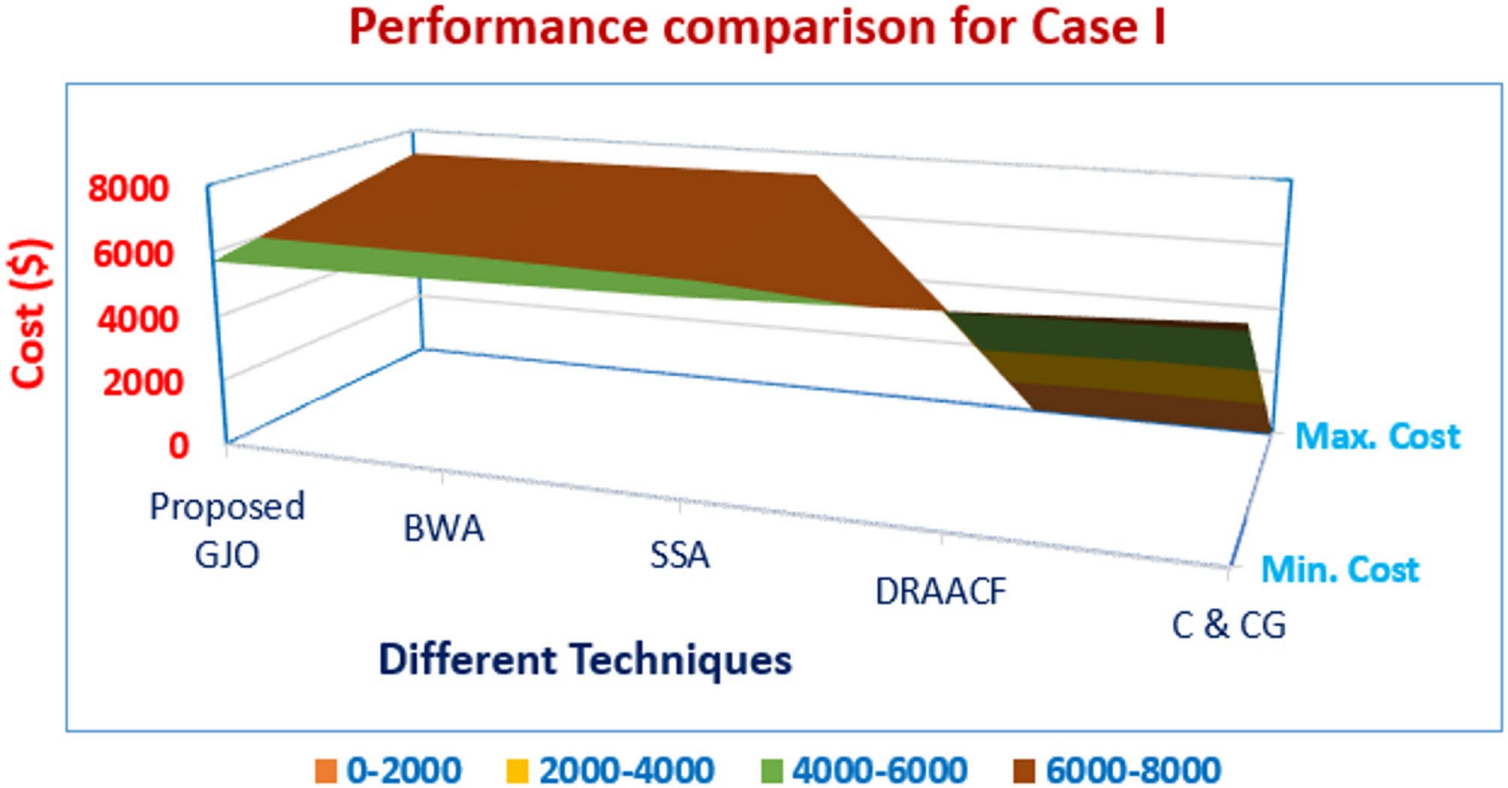
Performance comparison for Case I.

**Fig. 8:**
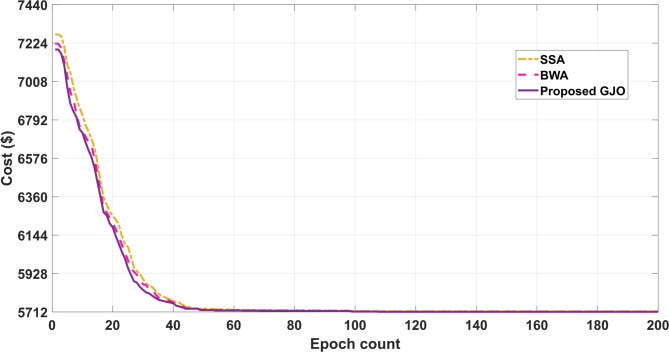
Convergence trend for Case I.

In Fig. 7, performance evaluation is represented based on the results attained from different techniques and it is observed that the solutions obtained from the proposed GJO outperform other methods. The resulting convergence for cost from different techniques is presented in Fig. 8.

#### Case II: single objective scheduling (minimization of emission)

In this case, emission reduction is carried out in a single objective framework as the emission is selected as an auxiliary objective function. The main purpose of including emission as a target objective function is due to the inclusion of CHP units in the current configuration of the VPP system under study. The emissions are minimized in this work along with the cost to promote a sustainable living and a pollution-free environment. Table 4 consists of the quality solution sets obtained by the optimization process in which the main parameters are minimum and maximum values of emission followed by mean emission. The results obtained are also confirmed with the reported work and it is observed that the proposed GJO method performs exceptionally well, especially in terms of computational time. The percentage reduction in emission from the proposed GJO method when linked with PSO by 24.5% and 13.57% when compared with the ε-constraint method. Also, there is a marginal improvement of 2.12% and 1.18% in the reduction of emissions when compared with other SSA and BWA.

**Table 4:** Performance assessment with employed techniques for Case II.

Output (Kg)	Proposed GJO	BWA	SSA	ε-constraint^38^	PSO^39^
Min. Emission	48,635.13	49,211.49	49,685.64	56,270	64,432.3217
Max. Emission	61,985.12	62,013.54	62,785.97	77,430	67,077.3937
Mean Emission	55,310.12	55,612.51	56,235.8	-	66,070.1682
Computational run time (sec.)	115.34	123.94	146.32	-	171.48

**Fig. 9:**
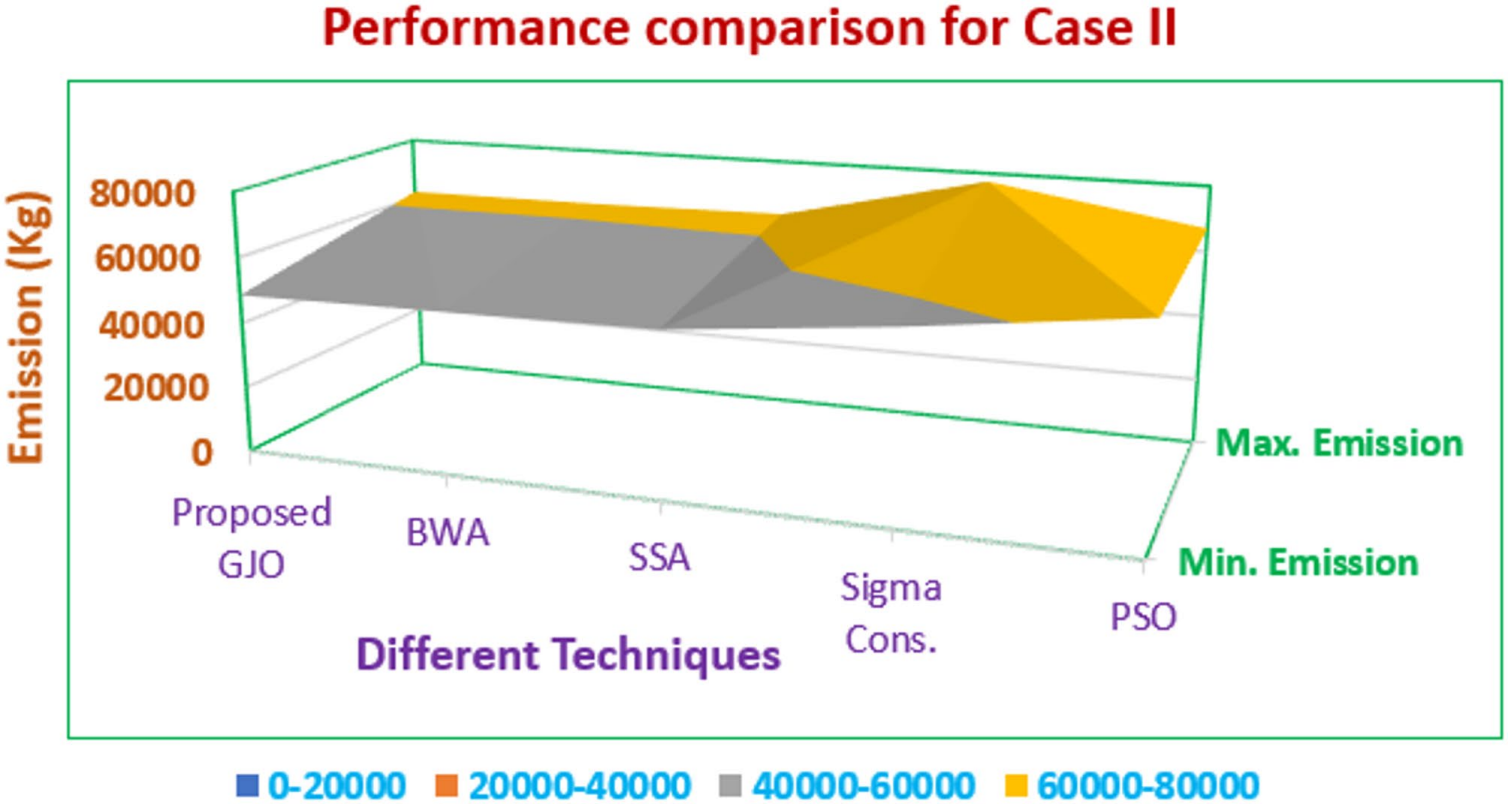
Performance comparison for Case II.

**Fig. 10:**
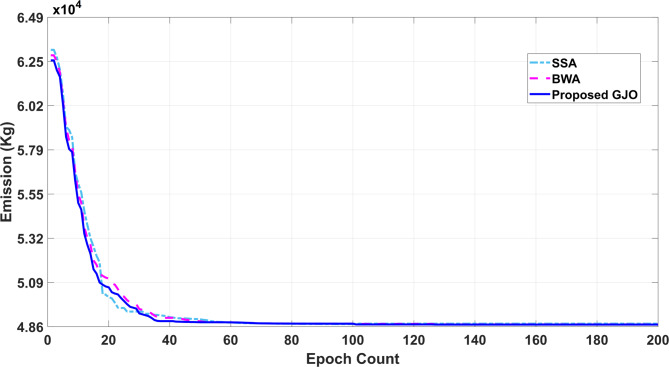
Convergence trend for Case II.

Quality solution sets obtained from multiple techniques and their performance in terms of minimum and maximum emission are represented in Fig. 9 in which GJO seems suitable over other methods. The convergence trend for emission is presented in Fig. 10 in which all the techniques are grouped for a holistic comparison.

#### Case III: multi-objective scheduling (minimization of cost/emission)

Finally, concurrent scheduling is executed in Case III in which the target objectives are minimized, i.e., cost/emission simultaneously in a multi-objective context. The comparison table is presented in Table 5 in which the numerical results are reported after making the simulation run for several independent runs. The outcomes presented in this case are also compared with the work available in the literature and a Pareto-based approach is also developed for the proposed formulation which becomes relevant when multiple objectives are involved due to their conflicting nature and non-linear characteristics.

**Table 5:** Comparison table for Case III.

Objectives	Cost ($)			Emission (Kg)		
Parameters	Proposed GJO	BWA	SSA	Proposed GJO	BWA	SSA
Min	3085.64	3175.68	3350.39	48,635.13	49,211.49	49,685.64
Max	5712.71	5748.42	5835.6	1,07,668.24	1,08,278.73	1,08,625.16

**Fig. 11:**
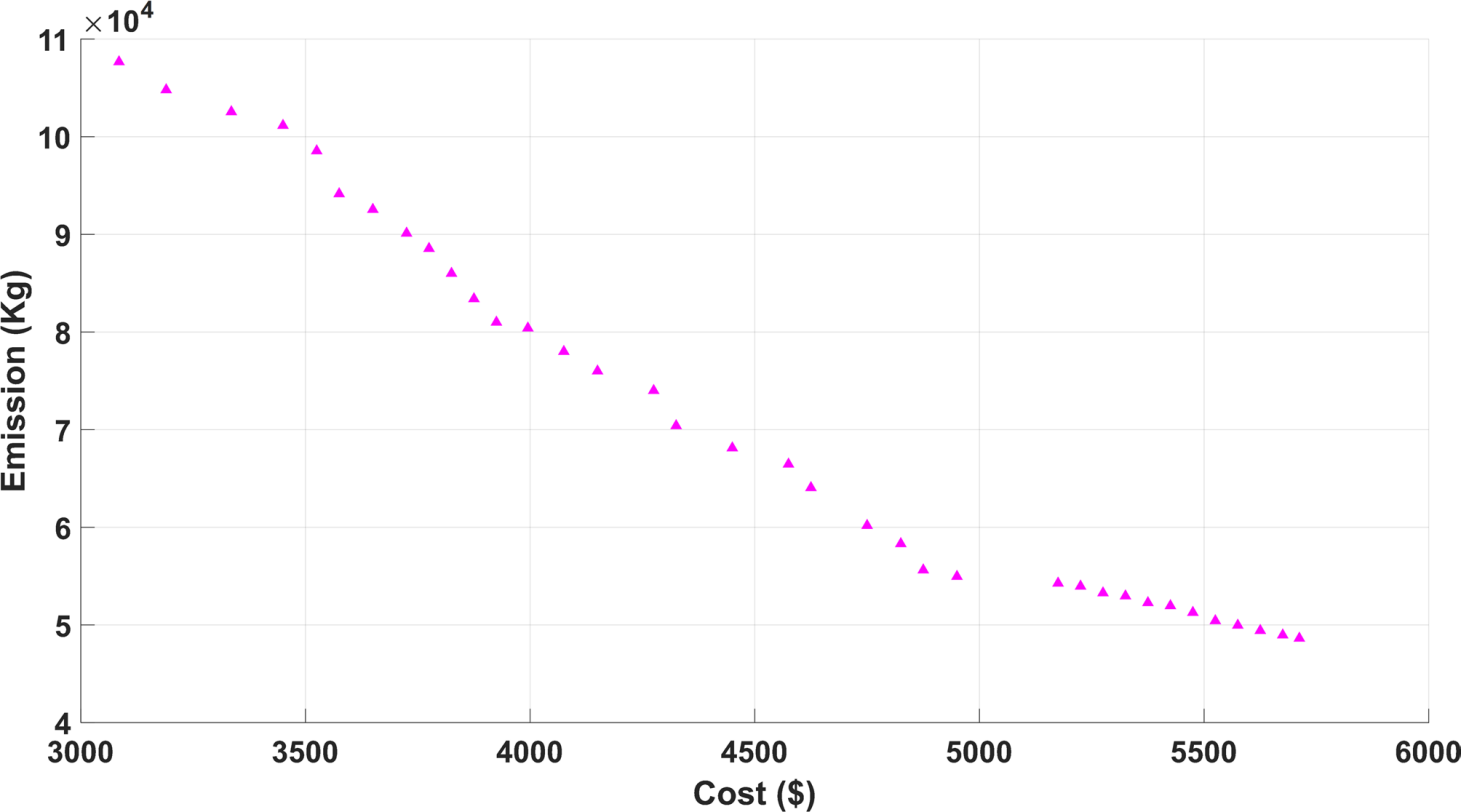
Pareto solution obtained from proposed GJO for Case III.

Figure 11 shows the Pareto optimal solution obtained from the proposed GJO in which objective 1 i.e., cost is presented on the X-axis and objective 2 i.e., the emission is on the Y-axis.

### Computational analysis

**Fig. 12:**
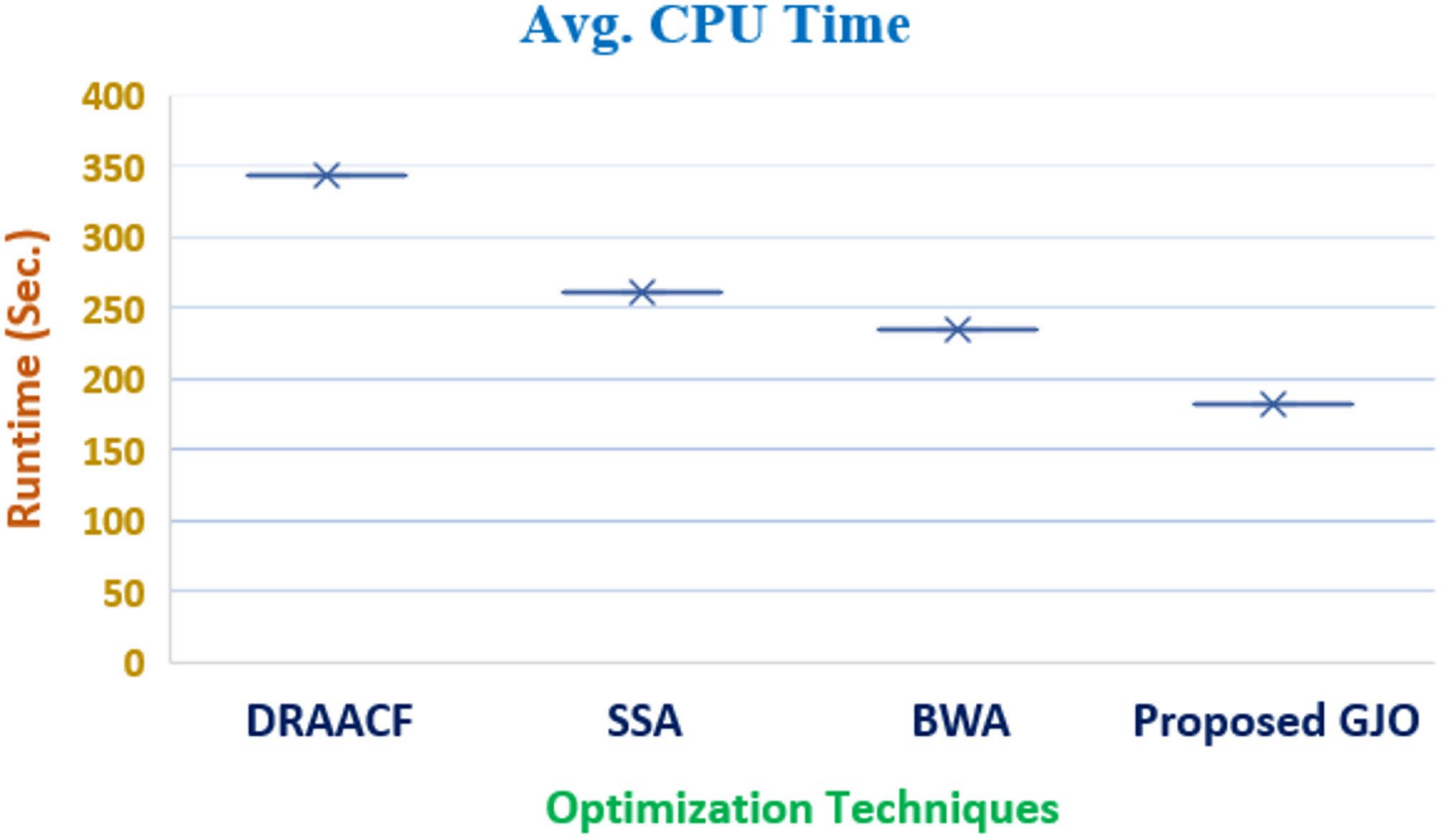
Elapsed time consumed to solve Objective 1.

**Fig. 13:**
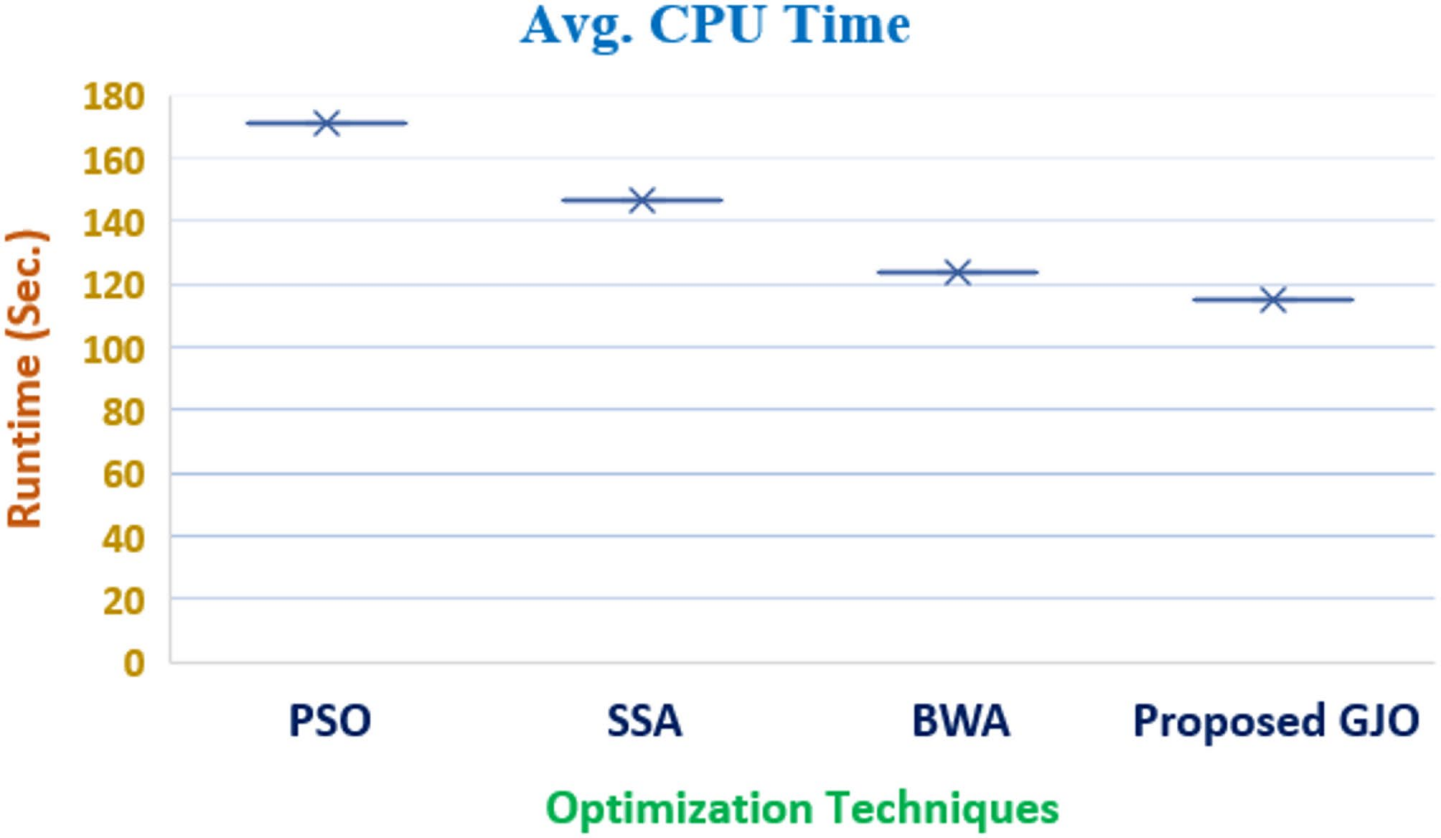
Elapsed time consumed to solve Objective 2.

The target objectives are solved by multiple techniques and three cases are discussed in which single and multi-objective solutions are presented. The computational time required to reach the desired solution become relevant while discussing the real-world engineering problem. It is observed that SSA and BWA consumed 261.46 and 235.21 secs, respectively for Case I which is better than time consumed by DRAACF which is 342.87. It is evident that proposed GJO consumes minimum time and outperforms other techniques by solving the desired objective in 181.97 s. The elapsed time taken by multiple techniques are shown in Figs. 12 and 13 for objective 1 and 2, respectively.

Also, for Case II, the time taken to attain the desired solution by PSO is 171.48, making it less efficient when correlated with the other techniques employed to solve the desired objective. The time consumed by SSA and BWA are 146.32 and 123.94, less than PSO. In addition, the time consumed by the proposed GJO is merely 115.34 s which validates its efficacy as compared to other techniques.

Since, the work carried out in this research deals purely in the VPP domain and an attempt has been made to demonstrate the system’s feasibility in practical scenarios. In support of this statement, we have performed an analysis for the day ahead scheduling which can be a genuine basis to schedule the power dispatch at the discretion of the VPP operator based on the availability of the associated resources in the studied configuration. Moreover, the real-world cases are also discussed in our latest study in which the scheduling is further scaled down on an hour ahead and real-time basis^40^. Therefore, the proposed approach can certainly be an effective option to maintain the power balance throughout the operation. The numerical analysis is performed for the VPP system configuration discussed in this work considering not only the economical aspect but also the environmental perspective. The approach is suitable for any real-time system by scaling down the optimization interval on hour-ahead scheduling as performed in^40^. Also, various ongoing projects in the VPP domain are already in the operational phase and the performance efficiency is also commendable. A few examples are included but not limited to Tesla where the VPP project is carried out in South Adelaide, Australia in line with the approval of the Australian Government which is a very successful operation that ends up benefiting nearly 50,000 tenants cumulatively^41^. Another major operation is carried out by a company based in California viz., Auto Grid Systems which is anticipated to be the World’s largest with context to the number of interconnected assets^42^. The VPP technology is getting better with every passing day and it is preferred by most nations to make the existing grid smarter and cleaner in a holistic manner. However, the power sector can benefit more from VPP technology by balancing supply and demand in real-time, managing the intermittencies of renewables, peak load management and finally promoting the participation in energy market.

The VPP theme is emphasized in this paper following which deep insights related to this technology are discussed. The complexity of the system is demonstrated in terms of a detailed workflow in which the aggregation of renewables is performed by the VPP operator and the problem is efficiently solved by utilizing a nature-inspired GJO-based optimization technique. The VPP technology is paving the way for further advancements in the energy sector by leveraging the true potential of the smart grid (SG) which also requires a robust infrastructure. Also, cost and emission reductions are considered as the target objectives to make the VPP more commercially and environmentally feasible. Nonetheless, there are a few technical limitations that exist i.e., the timid nature of utility operators, and limited awareness of the consumers turned prosumers followed by the policymakers as this technology involves software-based advancements. This may bring a few challenges like cybersecurity and a lot of data transfer and therefore, this issue will be taken care of in our upcoming work and the research direction is broadly open to the readers.

### Potential challenges and solutions

This work emphasizes the importance of VPP in the energy sector to escalate the smart grid technologies by leveraging their true potential to further advance 3Es i.e., energy, environment and economy. The VPP technologies rely on software-based innovations and therefore, the adoption of advanced optimization techniques is necessary to demonstrate the ability of the VPP system and eventually attain optimum results of the target objectives. However, commercial aspects of VPP are duly considered in this which is evident in the numerical analysis. The technical aspects are also taken care of to some extent i.e., power balancing by considering all the resources in the energy mix. Power balancing plays a crucial role in power systems operation and maintaining them throughout the operation is difficult yet necessary. The VPP operator is responsible for maintaining this management and control by initially aggregating the available power from all the resources and thereafter, dispatching the power in the most efficient way through intelligent distribution. Nonetheless, the VPP is an economically feasible technological advancement that the energy sector is witnessing yet there lies a challenge in the implementation phase. Several pilot projects are already in the operational phase and the feedback from both utility and prosumers is excellent. The private giant Tesla is a major example where the pilot project is carried out in South Adelaide, Australia in line with the nod of the Australian Government which is a very successful operation that ends up benefiting nearly 50,000 tenants cumulatively. Another major operation is carried out by a company based in California viz., Auto Grid Systems which is anticipated to be the World’s largest with context to the number of interconnected assets. These are a few examples in the domain of VPP and this technology will certainly mature soon to be accessible by most of the nations including India to make the existing grid smarter and cleaner in a holistic manner. The VPP-based system relies mostly on software-based inventions and therefore, the adoption of advanced optimization techniques is necessary to demonstrate the true ability of this technological innovation. The VPP operator is mainly responsible for maintaining holistic management and optimum control by initially aggregating the available power from available resources and thereafter, dispatching the power by initiating intelligent distribution. Nonetheless, the VPP is an economically and environmentally feasible technology witnessed by the energy sector and proper infrastructure is required for flawless operation. Since, the improvement in the existing grid structure is an ongoing process that cannot happen overnight but eventually to extract the maximum potential of SGs, the VPP is indeed going to play a key role. Several pilot projects are already in the operational phase and the feedback from both utility and prosumers is excellent. In particular, this technology will certainly be established due to its responsiveness and self-healing capabilities soon which will be accessible by the aspiring nations to be independent in the energy scene to make the existing grid smarter, cleaner and more resilient in a holistic manner.

### Discussion

There are various practical implications of a VPP and the idea of this technology excites the policymakers in the energy sector. Unlike microgrid (MG), VPP doesn’t have any political hindrance or any other regulatory restrictions which makes it more feasible and practically viable as VPP aimed directly at wholesale markets to perform energy-related transactions. The pricing mechanism is also defined accordingly considering both user convenience and utility operator’s discretion. Therefore, the accessibility of a VPP is deemed more as compared to any other potential alternatives which also include yet another important derivative demand response (DR) which is shaping the future power requirement more responsive and efficient. Nonetheless, the most known and broad category of VPPs is commercial-type VPP (CVPP) and technical VPPs (TVPP). In this work, the commercial aspect is more emphasized in which cost reduction is the primary objective along with cutting down the generated emission which is important from the environmental point of view. Thus, relatable cost–benefit analysis is discussed by satisfying both economic and environmental constraints. The proposed VPP problem is selected which consists of multiple resources including small hydro and co-generation units to increase the robustness of the DER network. The purpose is to reduce the cumulative cost linked with the system to make it economically feasible. The developed VPP problem is non-linear and caused by the sporadic nature of renewable-based sources i.e., solar and wind along with the variable market price. The system’s total cost is optimized by employing an advanced GJO technique which is promising in terms of rapid convergence characteristics and reduced elapsed time followed by providing the desired solution in a minimum number of epochs. The work performed encourages the participation of VPP in energy-related services to reduce the higher cost associated with the system and at the same time can encourage the utility operator to schedule the load demand in an optimized manner which is not only efficient but also economically viable. Moreover, the simplicity and lucrative computational efficiency are evident from the proposed approach and the decrease in cost and emission verifies the applicability of the operation performed by the developed VPP.

## Conclusion

The majority of renewable share from non-conventional sources in the energy mix is undoubtedly preferred to have a clean and greener ecosystem as the energy infrastructure plays a major role in the growth of any nation. The management and control of these renewable powered sources are essential to promote them to increase their energy share and to leverage their potential, a VPP system is proposed in this work. The following are the conclusive summary and highlights of the proposed work.

- This paper reports the optimal operation and scheduling of a VPP performed on a day-ahead (DA) and various resources are combined to form a network of DERs aggregated to maintain a demand and supply balance at any given time.
- Renewable-based sources i.e., Solar PV, wind power and fuel cell are selected along with small hydro units which increases the dependability of the system in case solar or wind is not able to fulfil the power requirement. In addition, multiple reserve options are provided for the proposed configuration of the VPP configuration under study in storage provision is provided.
- Moreover, to handle the non-linear characteristics associated with these resources, a recently developed and nature-inspired Golden Jackal Optimization is employed to solve the proposed problem formulation.
- This work solves dual problems selected as target objectives in which minimization of cost/ emission are performed initially in a single objective context. Furthermore, the proposed formulation is then extended in a multi-objective framework in which simultaneous reduction of system cost and generated emission are performed.
- The percentage reduction in the cost from the proposed GJO is better when compared with other techniques i.e., SSA and BWA by 2.1% and 0.62% followed by the decrease in emission from the proposed GJO method when linked with SSA and BWA by 2.12% and 1.18%, respectively.
- Finally, the results are discussed and the quality solution sets are linked extensively with the reported work and the outcome reveals the suitability and effectiveness of the developed approach.

The present approach can be further extended in the future by incorporating hybrid systems resources i.e., hydro-thermal or additional storage option i.e., pumped hydro storage system which can further advance the consistency and performance of the system. Also, the inclusion of more resources will pose other challenges which can be solved by utilizing a hybrid variant of the optimization technique to deal with the complex nature of the problem. Therefore, seeing the system’s preparedness and responsiveness in a more complex framework would be interesting.

## Electronic supplementary material

Below is the link to the electronic supplementary material.

Supplementary Material 1

## Data Availability

The datasets used and/or analysed during the current study available from the corresponding author on reasonable request.

## Abbreviations

RERs: Renewable energy resources
VPP: Virtual power plant
EMS: Energy management systems
EM: Energy market
EP: Electricity pricing
RE: Renewable energy
ATC: Aggregated technical and commercial losses
DR: Demand response
DERs: Distributed energy resources
EVs: Electric vehicles
DA: Day-ahead
RT: Real-time
MG: Microgrid
GJO: Golden Jackal optimization
BWA: Beluga whale algorithm
BWs: Beluga whales
SSA: Salp swarm algorithm
ESUs: Energy storage units
SH: Small hydro
C&CG: Column and Constraint Gradient
CHP: Combined Heat and Power
SG: Smart grid

## Code availability

The code is provided which is developed by the authors as a part of this study.
